# Regulation of morphogen signaling pathways by recurrent hypermotif circuits

**DOI:** 10.1073/pnas.2538060123

**Published:** 2026-10-05

**Authors:** Miri Adler, Ruslan Medzhitov

**Affiliations:** ^a^ Department of Genetics, Silberman Institute of Life Science, Edmond J. Safra Campus, The Hebrew University of Jerusalem, Jerusalem 9190401, Israel; ^b^ Department of Immunology and Cancer Research, Faculty of Medicine, The Hebrew University of Jerusalem, Jerusalem 9112102, Israel; ^c^ Tananbaum Center for Theoretical and Analytical Human Biology, Yale University School of Medicine, New Haven, CT 06510; ^d^ Department of Immunobiology, Yale University School of Medicine, New Haven, CT 06510; ^e^ HHMI, Yale University School of Medicine, New Haven, CT 06510

**Keywords:** gene regulatory networks, morphogens, network motifs

## Abstract

Morphogen signaling pathways orchestrate cell fate and tissue patterning during embryo development through intricate intercellular communication. While much is known about the molecular components of these pathways, the underlying regulatory logic shaping their integration remains unclear. Here, we develop a computational framework to uncover higher-order network structures, *hypermotifs*, that link gene regulatory circuits within and between cells via morphogen signaling. Applying this framework to single-cell RNA sequencing data from human intestinal development, we identify recurrent hypermotif topologies that mediate communication between epithelial and fibroblast populations. These cell-cell hypermotif circuits involve feedback and feedforward loops embedded within signaling pathways. Dynamical modeling reveals that such circuits can generate emergent behaviors, including oscillatory and antagonistic responses. Our results highlight hypermotifs as key design principles in developmental regulatory networks, providing a systems-level understanding of morphogen signal integration across cell types.

## Significance

Tissues are patterned by signaling molecules (morphogens) that cells both sense and produce, but the regulatory logic connecting morphogen response to morphogen production across different cell types is poorly understood. We develop a framework that links intracellular gene-regulatory circuits to intercellular communication, enabling systematic discovery of recurring cell-cell circuit architectures during development. Applied to epithelial-fibroblast interactions, the method reveals repeated circuit designs that coordinate multiple signaling pathways. Using mathematical modeling, we show these designs can naturally generate complex but interpretable behaviors, including pulses, oscillations, and opposing responses, providing a mechanistic explanation for how tissues reliably integrate signals to organize in space and time.

**Fig. 1.**
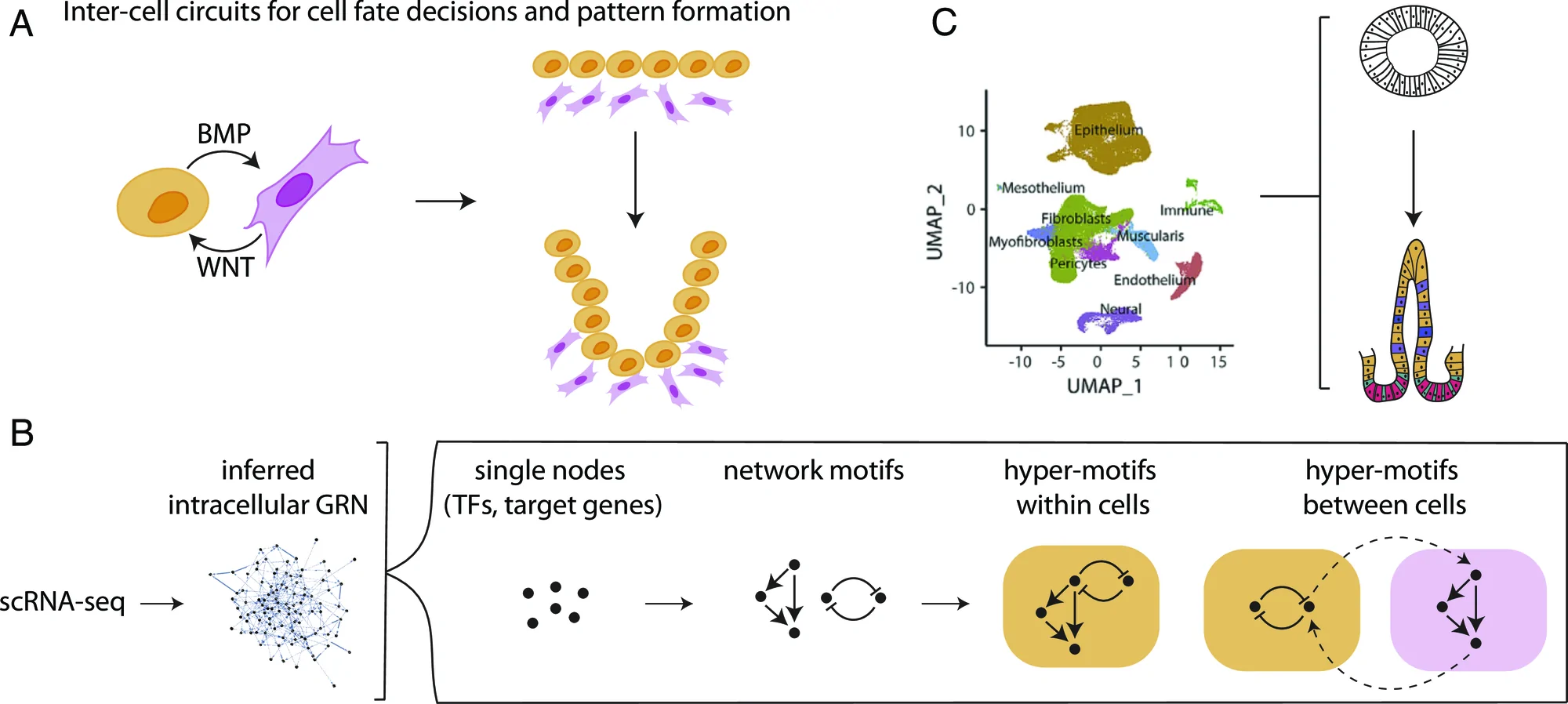
Analyzing intercellular circuits that integrate regulatory interactions using the hypermotif framework. (*A*) What are the design principles of intercellular circuits in developmental programs for cell-fate decisions and pattern formation? (*B*) We infer and study intercellular circuits in developmental programs by analyzing network motifs in scRNA-seq-derived gene regulatory networks and how they combine within and between interacting cells through morphogen signaling. (*C*) We apply our approach to scRNA-seq data from Fawkner-Corbett et al. (32) that span the development of the human intestine.

In multicellular organisms, the intricate processes of cell fate decisions and tissue patterning are driven not only by internal cellular mechanisms but also by intercellular communication (1). This communication is mediated through morphogens, key signaling molecules that diffuse across tissues, forming gradients that dictate developmental pathways for cells (2–6). These morphogen gradients provide essential spatial information that cells utilize to properly organize and differentiate during embryogenesis (Fig. 1*A*). Important morphogen families, such as bone morphogenic proteins (BMPs) (7, 8), fibroblast growth factors (FGFs) (9), Hedgehog (HH) (10, 11), WNT (12, 13), and Retinoic acid (14), form a complex network together with their receptors, coreceptors, and antagonists. These interactions are regulated at multiple levels, including posttranslational modifications, protein-protein interactions, and regulatory circuits that are orchestrated by key developmental transcription factors including HOX, GATA, ETS, SOX, PAX, TBOX, FOX, and E-proteins (15–23). Together, these elements form an intricate network that governs cellular behavior and tissue architecture (24–31).

Despite significant progress in deciphering morphogen signaling pathways, the underlying principles of morphogen signal integration at both cellular and tissue levels are not fully understood (33). This includes how transcriptional circuits within cells and the regulation of morphogen ligands, receptors, and coreceptors among cells contribute to developmental dynamics. To address this, we developed a computational framework to investigate how building-block regulatory circuits within cells are integrated with ligand-receptor interactions between cells during embryonic development.

Previous studies have identified network motifs as fundamental components of regulatory networks (34–37). Yet, the integration of these motifs within and among cells through morphogen signaling remains largely unclear. We have recently introduced hypermotifs, higher-level network modules that describe how individual motifs are incorporated within broader networks (38). Hypermotifs are defined as multiple network motifs that either share at least one node (combinations of motifs) or are directly linked through regulatory interactions (motif interactions) (Fig. 1*B*). These higher-level structures can form hypermotif regulatory circuits within cells, which we elaborate on in an accompanying study in Adler and Medzhitov (39), or hypermotif circuits between communicating cells, linked by morphogen signaling pathways, which is the primary focus of this study. Using this approach, we dissect and analyze how network hypermotifs, linking transcription factors and signaling molecules, form complex configurations of cell-cell circuits. This framework uncovers the fundamental properties of intertwined regulatory circuits, enhancing our understanding of cellular communication and decision-making during embryogenesis and clarifying the emergent properties of developmental regulatory networks.

## Results

### Analyzing Developmental Gene Regulatory Networks in Human Intestinal Development Using Single-Cell Transcriptomics Data.

To develop our framework, we analyzed single-cell RNA sequencing (scRNA-seq) data from Fawkner-Corbett et al. (32), a single-cell atlas of the developing human intestine spanning nine developmental stages, from 8 to 22 postconception weeks (PCWs) (Fig. 1*C*). In our accompanying study, we used these data to infer gene regulatory networks comprising developmental transcription factors (TFs), morphogen ligands, their antagonists, receptors, and coreceptors (*SI Appendix*, Table S1). These SCENIC-inferred GRNs represent predicted transcriptional regulatory interactions. To reduce sensitivity to stochastic variation in network inference, the SCENIC pipeline was repeated six times and overlapping regulons were retained. We examined these networks at each time point, identifying their most statistically enriched network motifs and hypermotifs. We identified five network motifs consistently present throughout development: autoregulation (mostly positive), mutual feedback loops, regulated feedback loops, regulating feedback loops, and feedforward loops (FFLs). Employing a computational method we recently developed, we analyzed how these motifs combine within the network and identified prevalent motif combinations. These hypermotif structures reveal common patterns: autoregulation often connects with feedback circuits or FFLs at their intermediate and output nodes, while feedback circuits typically link to FFLs via their intermediate nodes. Moreover, motifs frequently share a common input or output node. Most of these hypermotif structures are specifically enriched at 10 PCW, approximately the time at which the crypt-to-villus axis is formed. Next, we investigate how these enriched hypermotif regulatory circuits within cells regulate morphogen expression.

### Cell-Cell Hypermotif Circuits Emerge Through Morphogen Signaling Pathways.

**Fig. 2.**
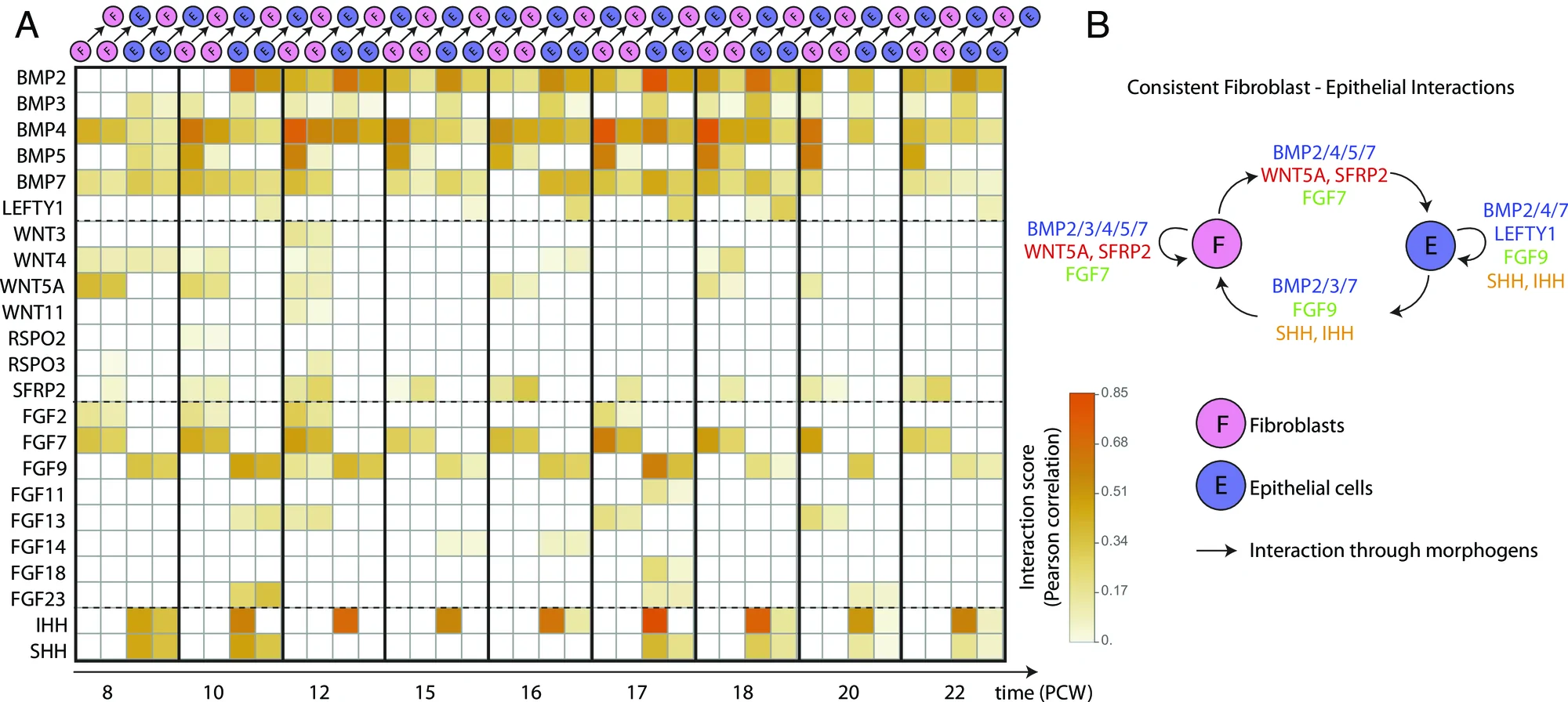
Cell-cell communication patterns among epithelial and fibroblast populations through morphogen signaling. (*A*) Heatmap of potential ligands based on epithelial (E) and fibroblast (F) differentially expressed genes along the developmental process. Ligand interaction scores (Pearson correlation) are presented by white-orange scale. (*B*) Cell-circuit between E and F cell type populations through morphogen and growth factor (GFs) provision from 8 to 22 PCWs that includes consistent interactions that received high interaction score in at least five time points. Black arrows from cells to GFs represent ligand production. Black arrows from GFs to cells represent binding to cognate receptors.

Using the gene regulatory networks we inferred, we explore how regulatory circuits within cells are linked through morphogen signaling pathways, creating hypermotif circuits between communicating cells. For cell communication circuit analysis, we focus on fibroblast and epithelial cell type populations, the two cell types playing important roles in governing intestinal development (40). First, we identify pairs of interacting cells where we consider interactions through ligand-receptor pairs of developmental morphogen families including BMP, WNT, HH, FGF, and retinoic acid. To identify pairs of interacting cells, we used Nichenet, a computational method that infers ligand-receptor interactions considering expression patterns of ligands, receptors, and downstream signaling genes in the receiving cells (41) (*Methods*). Nichenet predicts ligands that may explain the expression of downstream target genes in receiver cells by integrating ligand/receptor expression with prior knowledge of signaling and gene-regulatory interactions. The interaction score shown in Fig. 2*A* is the Nichenet ligand activity score (computed based on a Pearson correlation score) used to rank ligands according to how well their predicted target genes match the observed receiver-cell expression program. These inferred interactions should therefore be interpreted as candidate ligand-receptor links rather than direct measurements of ligand secretion or receptor activation. Using Nichenet, we highlight the top ligands that are predicted to link specific populations of fibroblasts and epithelial cells (Fig. 2*A*). Throughout the developmental process, we find that fibroblasts and epithelial cells communicate reciprocally through various BMP ligands; whereas IHH and SHH production is restricted to epithelial cells and its receptors and antagonists are produced by fibroblasts. Morphogens from the WNT and FGF families also show consistent patterns whereas several morphogens (e.g., WNT3, FGF11) appear with high interaction scores at specific time points (Fig. 2 *A* and *B*).

**Fig. 3.**
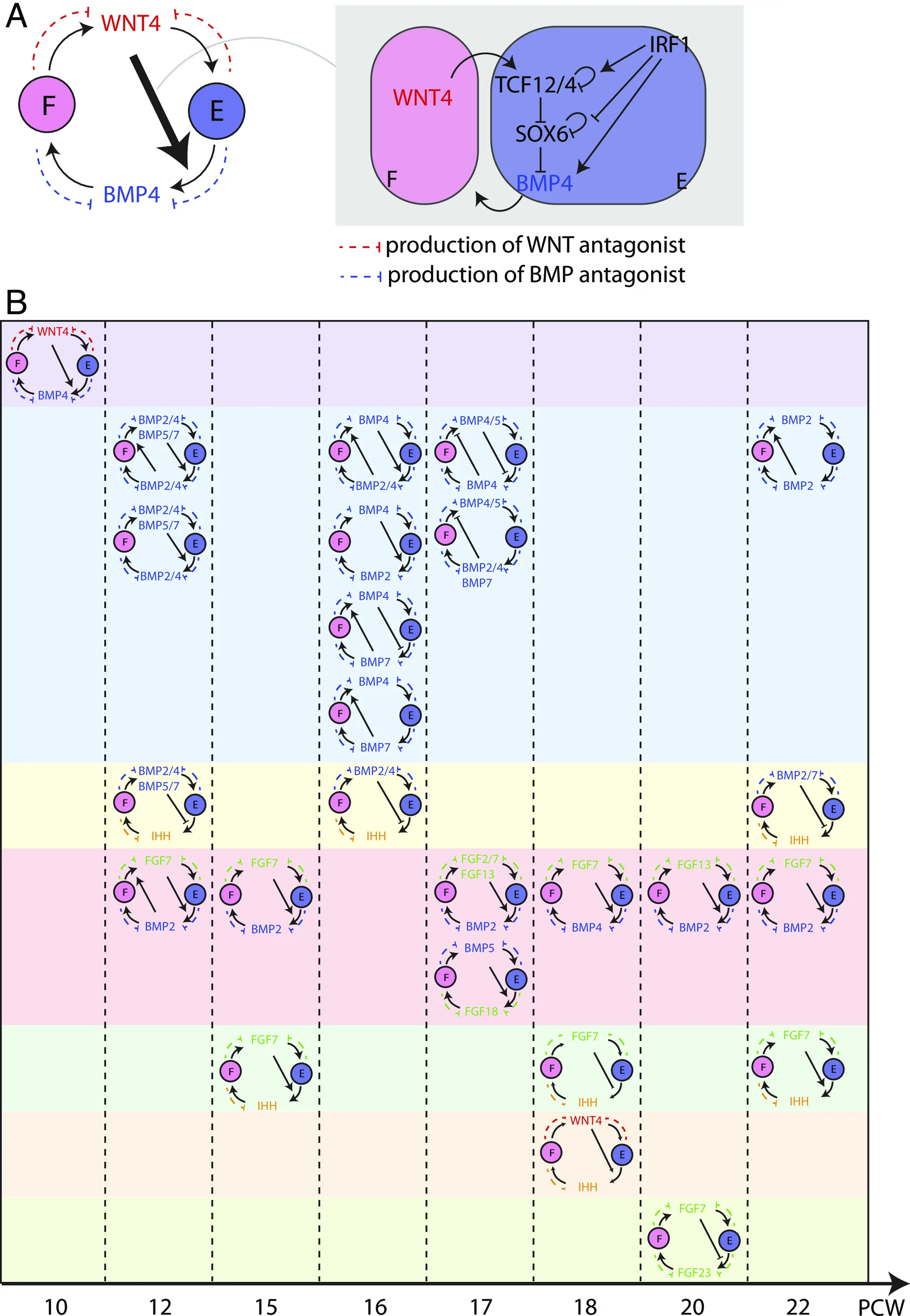
Inferring hypermotif circuits between communicating cells through ligand-receptor interactions. (*A*) Example of an inferred hypermotif circuit through WNT4 and BMP4 production at 8 PCW where expression of morphogen ligands is directly regulated by a regulatory circuit downstream to a morphogen signaling pathway. Black arrows from cells to GFs represent ligand production. Black arrows from GFs to cells represent binding to cognate receptors. Dashed colored inhibitory arrows represent inhibition of GFs through antagonist expression. The black arrow pointing from WNT4 to the production arrow of BMP4 represents upregulation through a gene regulatory circuit that is downstream to WNT4’s signaling pathway in epithelial cells as illustrated in the panel to the right. (*B*) Table of cell circuits inferred considering reciprocal morphogen provision among fibroblast and epithelial cell types across the intestinal developmental process. In every cell circuit, black arrows pointing from GF1 to another GF’s production arrow, GF2, represent activation or inhibition through a gene regulatory circuit that is downstream to the GF1 signaling pathway in the cell type producing the GF2 signal.

Next, we use these cell communication circuits to infer regulatory networks linking perception of certain morphogens to production of other morphogens. We focus on reciprocal circuits composed of provisions of pairs of signaling pathways. Out of these circuits, we look for situations where gene regulatory interactions that are downstream to a certain morphogen signaling pathway in the receiving cell directly upregulate or downregulate expression of a morphogen ligand (Fig. 3*A*). Following these morphogen circuits over time, we find several patterns that recur in multiple time points (Fig. 3*B*). For example, downregulation of IHH by BMP signaling appears at 12, 16, and 22 PCWs.

### Cell-Cell Hypermotif Circuits Can Provide Emergent Dynamical Properties.

**Fig. 4.**
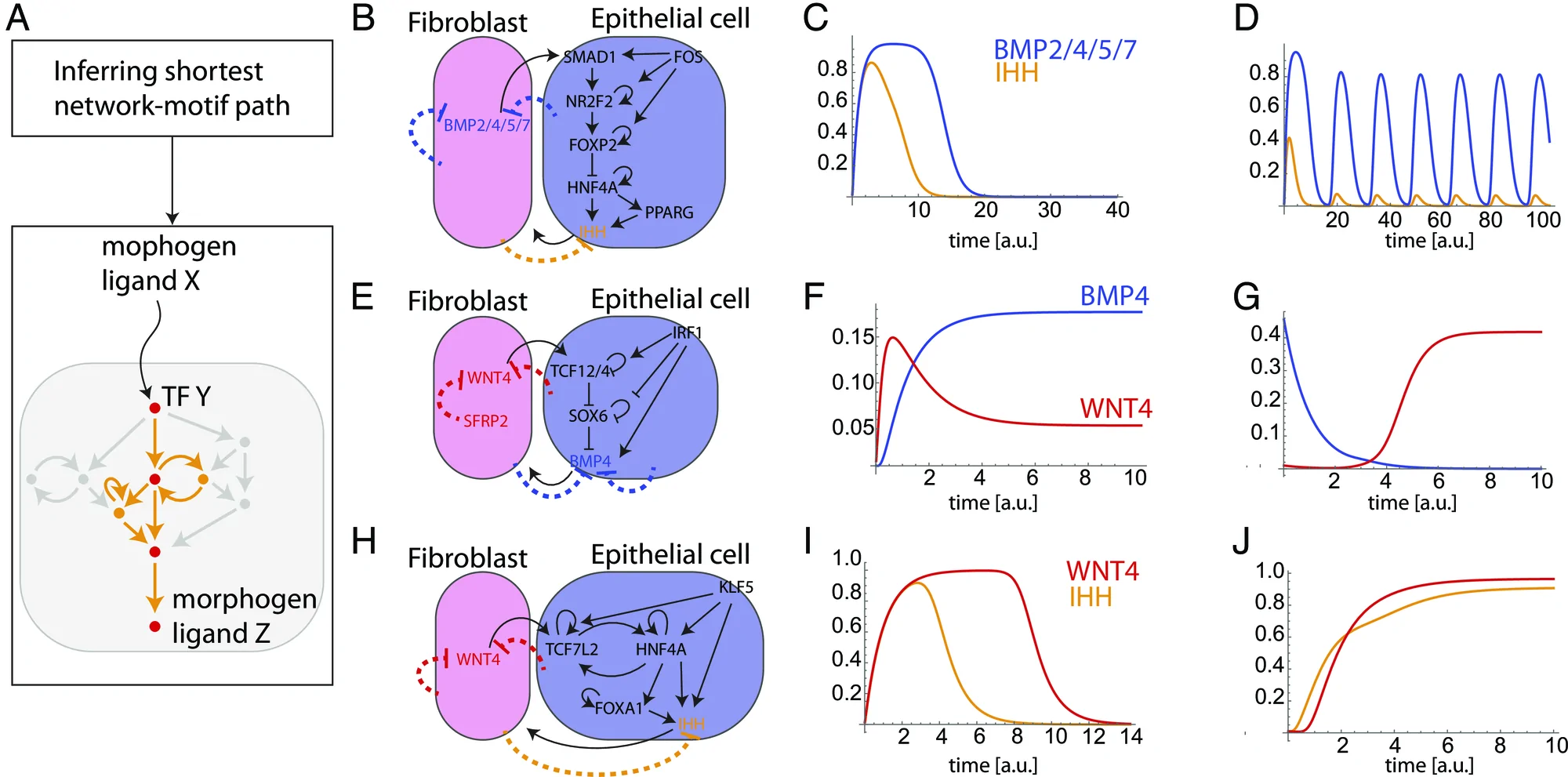
Dynamical properties of cell-cell hypermotif circuits. (*A*) Illustration of our method to infer the shortest network-motif path linking input signal X and output gene Z. Nodes participating in the shortest path are marked in red. Edges and additional nodes participating in the shortest network-motif path are marked in orange. (*B*–*J*) Examples of three hypermotif gene regulatory circuit topologies (*B*, *E*, and *H*) linking two morphogen signaling pathways between fibroblast and epithelial cells. Dashed arrows represent production of morphogen antagonists and are incorporated through effective morphogen turnover, availability, degradation, or inhibitory terms in the ligand equations. (*C*, *D*, *F*, *G*, *I*, and *J*) Examples of simulated dynamical behavior of the circuits in *B*, *E*, and *H* with varying parameters or initial conditions, using the parameter sets described in *Methods*. The simulations illustrate selected qualitative behaviors supported by each topology rather than an exhaustive analysis of all possible parameter regimes.

We consider three examples of hypermotif regulatory circuits between communicating cells and examine their potential dynamical behavior in more detail. To do so, we consider the shortest path in the gene regulatory network linking the TF (Y) downstream to the input morphogen signaling pathway (X) received by the cell and the output morphogen ligand (Z) that is produced by the same cell (Fig. 4*A*). We use the shortest path to represent the most direct intracellular regulatory route from morphogen sensing to morphogen production. This path may capture relatively immediate transcriptional effects because it includes the fewest inferred regulatory steps between the input pathway and the regulated morphogen gene. Since the genes participating in this shortest path are part of a larger complex gene regulatory network, we developed a method to extract the circuit topology controlling the expression of the output gene Z. Instead of simply considering a cascade of genes following the shortest path between X and Z in the network, we infer the “*shortest network motif path*.” Using the shortest network-motif path approach, we follow the network motifs in which the genes linking X and Z participate in and include the local motif context surrounding the shortest path (Fig. 4*A*, *Methods*). Thus, the extracted circuit represents a minimal and interpretable regulatory route together with the motif architecture in which this route is embedded. Longer parallel paths may also influence the full circuit dynamics, but may reflect more indirect, delayed, or context-dependent regulatory effects.

After inferring the shortest network-motif path between a pair of morphogen signals, we developed a mathematical model to explore possible dynamical behaviors of the resulting hypermotif circuit (*Methods*). Dashed inhibitory arrows indicate morphogen antagonism inferred from antagonist expression. In the reduced ODE models, these effects are represented through effective morphogen availability, turnover, degradation, or inhibitory terms rather than as separate antagonist variables (*Methods*). The simulations illustrate selected qualitative behaviors that can be supported by the inferred topologies. We find that interactions between members of BMPs and IHH (Fig. 4*B*) can provide pulsatile response (Fig. 4*C*) and pure oscillations (Fig. 4*D*). Interactions between BMP4, WNT4, and its inhibitor SFRP2 (Fig. 4*E*) can yield antagonistic expression patterns for BMP4 and WNT4 depending on model parameters (Fig. 4 *F* and *G*). The hypermotif in epithelial cells that regulates IHH and is activated by WNT4 (Fig. 4*H*) can provide a pulsatile (Fig. 4*I*) or delayed and sustained (Fig. 4*J*) response. Other behaviors may occur under different parameter choices, and quantitative prediction of a specific in vivo circuit would require a more detailed model including explicit antagonist production, ligand binding, receptor activation, and diffusion.

### Hypermotif Regulatory Circuits Frequently Regulate Morphogen Expression.

**Fig. 5.**
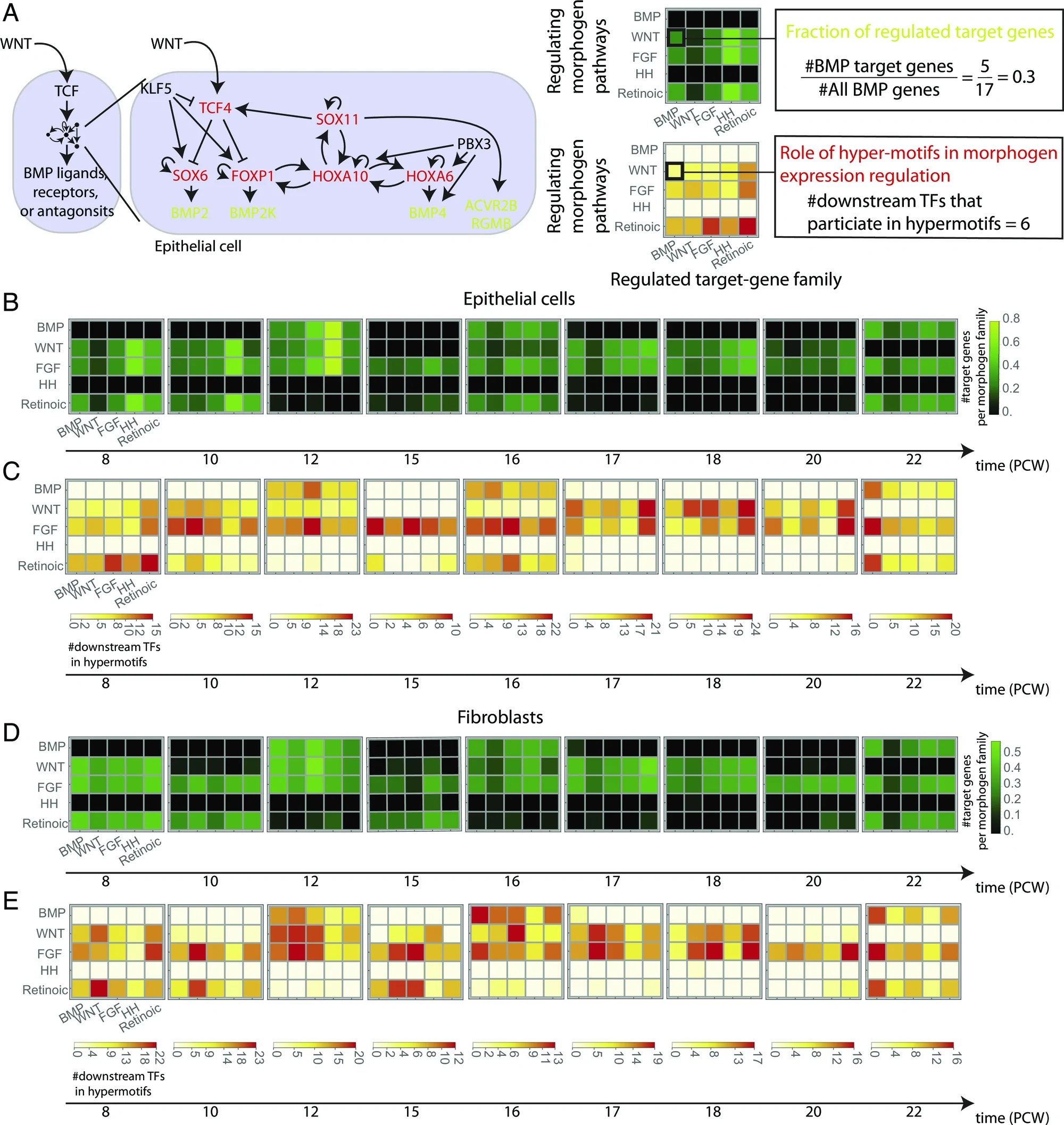
Role of hypermotif circuits in regulating morphogen signaling pathways. (*A*) An example of regulatory interactions in epithelial cells at 8 PCW that regulate BMP target genes downstream of WNT signaling. The small heatmaps illustrate the row/column convention used throughout the figure: rows represent the regulating morphogen pathway, and columns represent the regulated target-gene family, including ligands, receptors, and antagonists. (*B*) Heatmap of the fraction of target genes that are regulated downstream of morphogen signaling pathways in epithelial cells. Each 5 × 5 heatmap corresponds to one developmental time point. A square in X row and Y column is colored by the number of ligands, receptors, and antagonists belonging to morphogen family Y that are found to be directly regulated in response to morphogen ligands from the X family, divided by the total number of genes assigned to the Y family. (*C*) Heatmaps of the number of genes in the regulatory circuits described in (*B*) that participate in more than one motif role (forming a hypermotif) in epithelial cells. (*D*) Heatmap of the fraction of target genes that are regulated downstream of morphogen signaling pathways in fibroblasts. (*E*) Heatmaps of the number of genes in the regulatory circuits described in (*D*) that participate in more than one motif role (forming a hypermotif) in fibroblasts. Rows and columns are defined as in (*A*).

Next, we explore the extent to which hypermotif circuits regulate expression of morphogens, their receptors, and antagonists. For every pair of morphogen families, we examine whether there is a direct path in the gene regulatory network linking one morphogen signaling pathway to the expression of another morphogen’s ligand, receptor, or antagonist (Fig. 5*A* and *SI Appendix*, Table S2). Counting the number of target genes belonging to a certain morphogen family that are regulated by another morphogen signaling pathway show large variation between the pairs of morphogens, as well as variation in the pattern over time (Fig. 5 *B* and *D*). Additionally, we explore the number of genes, within the regulatory paths linking pairs of morphogen signaling pathways, that participate in network hypermotif circuits. We find that most regulatory paths linking morphogen pairs are enriched with hypermotif regulatory circuits (Fig. 5 *C* and *E*). Interestingly, the overall patterns of morphogen-to-morphogen regulation are relatively similar between epithelial cells and fibroblasts, and show similar temporal changes across development (Fig. 5 *B*–*E*). This similarity suggests that epithelial and fibroblast cells may participate in a shared tissue-level morphogen signaling architecture during intestinal development, while implementing this regulation through distinct cell-type-specific ligands, receptors, antagonists, and intracellular GRN contexts. Examining the composition of the most common hypermotif regulatory circuits within these paths, we find that many of the regulatory circuits regulating expression of morphogen signaling correspond to the most statistically enriched hypermotifs we previously detected in the networks, suggesting that these enriched hypermotif circuits may contribute to regulating morphogen expression patterns.

## Discussion

Tissue patterning during embryonic development is a well-orchestrated process largely governed by morphogen signaling pathways, which coordinate cell fate decisions through tightly regulated intercellular communication. In this study, we introduce a framework for analyzing cell-cell communication where we integrate *hypermotif* circuits, composite network motifs embedded in transcriptional regulatory networks that are connected by morphogen signaling among communicating cells. By integrating single-cell transcriptomic data with known ligand-receptor interactions, we systematically infer how gene regulatory motifs within cells are linked to form larger cross-cellular regulatory structures. Notably, we observe that epithelial and stromal cells engage in temporally dynamic yet recurrent communication patterns mediated by morphogens such as BMP, WNT, and IHH. These interactions are often embedded within hypermotif architectures, suggesting a conserved regulatory grammar for morphogen coordination during intestinal development.

Beyond structural mapping, our analysis suggests that hypermotif circuits can provide nontrivial dynamic properties, including oscillations, pulses, and bistable switches. These emergent behaviors are important for generating temporal patterns of gene expression and robust developmental outcomes. Hypermotifs may therefore provide an intermediate scale of description between individual regulatory interactions and whole gene regulatory networks. This intermediate scale could be useful for prediction, because recurrent hypermotif architectures identify circuit structures with modelable dynamical capabilities, such as buffering, oscillations, pulses, bistability, or antagonistic pathway regulation. This approach can therefore provide a basis for building predictive models of cellular behavior, offering a means to investigate how cell communication shapes tissue organization. In addition, hypermotif analysis may help guide experimental control of developmental systems by highlighting regulatory nodes whose perturbation is expected to alter communication between morphogen pathways or change circuit dynamics. In organoids or engineered developmental systems, recurrent hypermotif architectures could also serve as design templates for constructing or modifying signaling circuits with desired behaviors. However, the dynamical behaviors modeled here should be interpreted as qualitative examples enabled by the inferred circuit topologies, rather than quantitative predictions of in vivo dynamics. Realizing predictive, control, or design applications will require experimental parameterization and validation of the inferred circuits.

The present analysis focuses on human intestinal development, and it remains to be determined whether the specific cell-cell hypermotif architectures identified here recur across other developmental contexts. Thus, the generalizability of this study lies primarily in the framework, whereas the extent to which the specific hypermotif architectures identified here represent universal morphogen-coupling circuits remains an open question. Applying the same pipeline to additional developing organs, ideally with matched time-resolved single-cell and spatial data, would help distinguish conserved cell-cell hypermotif architectures from tissue-specific implementations of morphogen signaling. In addition, linking these motifs to experimentally perturbed targets could reveal new strategies for manipulating developmental programs or correcting dysregulated signaling in disease contexts.

### Limitations of Study.

In this study, we focused on gene expression data and did not account for protein-level regulation or posttranscriptional modifications that play critical roles in morphogen signaling dynamics. As such, inferred regulatory circuits may miss key modulators or overestimate the functional impact of certain interactions. Because the scRNA-seq data provide static transcriptional snapshots, they do not uniquely constrain kinetic parameters in the ODE models; direct validation of the predicted dynamics will require targeted perturbations followed by time-resolved measurements of morphogen pathway activity and target-gene expression. In addition, while our framework captures topological and dynamical aspects of cell-cell communication, it does not yet incorporate spatial information or physical tissue architecture, which are essential for fully understanding morphogen gradient formation and signal propagation.

## Methods

### Inference of Cell Communication Circuits Through Morphogen Ligand-Receptor Interactions.

To infer interactions between epithelial and fibroblast populations, we applied Nichenet, an algorithm that infers ligand-receptor interactions within a transcriptomic dataset, using prior knowledge on ligand-receptor interactions and regulatory networks (41). We applied NicheNet to the intestinal development scRNA-seq dataset considering interactions between the specific cell type populations of fibroblasts and epithelial cells across the developmental process. The pairwise interaction between cell types (mature enterocytes, fibroblast progenitors, absorptive cells, etc.) was calculated independently for each time point in the data (nine time points spanning 8 to 22 PCWs). For each cell type and for each time point, genes of interest (GOIs) were defined as genes that were upregulated in a particular cell type compared to all other cell types in that time point and expressed in at least 25% of the cells within the cell type population. Interaction score was calculated as the Pearson coefficient calculated by “predict_ligand_activities” function, using the GOIs of that time point and the genes that were expressed in the “receiver” clusters as targets. For each pair of sender-receiver cells, we chose the top 10% quantile ligands. This analysis allowed us to infer interactions between every pair of specific cell type populations among the different fibroblast and epithelial clusters. In Fig. 2*A* we consider the possible interactions through the different morphogen ligand-receptor pairs where we group together the various fibroblast and epithelial clusters.

### Analysis of Shortest Network-Motif Paths.

To infer regulatory interactions that link a signal (S) perceived by a cell to an expression of a ligand encoded by a target gene (Z), we consider the GRN of that particular cell type. We use the shortest directed path as a minimal representation of the intracellular regulatory route linking morphogen sensing to morphogen-gene expression. This choice provides an interpretable starting point for extracting cell-cell hypermotif circuits, while longer parallel paths may also contribute to the full circuit behavior. We then use the function “FindShortestPath” in Mathematica to compute the shortest path linking a transcription factor X, which is downstream to the S signaling pathway and the target gene Z. Only directed paths following the inferred regulatory-edge direction were considered. Let us denote the set of genes that are in this shortest path $\left\{G_{s}\right\}$. We now build a new set of genes that will make up the shortest network-motif path: $\left\{G_{M}\right\}$. This procedure expands the shortest path by including the local network-motif context of the genes along the path, rather than treating the path as a simple linear cascade. For each gene $g_{i}$ in $\left\{G_{s}\right\}$, we perform the following steps:

Check if $g_{i}$ participates in network motifs in the same time point and in the same cell type.

If the answer to #1 is yes - add $g_{i}$ and the genes interacting with $g_{i}$ in the network motif to $\left\{G_{M}\right\}$. *Note that in case $g_{i}$ participates in multiple network motifs of the same topology and in the same motif role, we consider only one instance of this motif in $\left\{G_{M}\right\}$. This is similar to having multiple shortest paths in a network, and arbitrarily choosing one of them.

If the answer to #1 is no - add only $g_{i}$ to $\left\{G_{M}\right\}$.

The resulting shortest network-motif path therefore contains the most direct inferred regulatory route from S to Z together with the motif structures in which the path genes participate. Regulatory paths and motif structures extracted in this way are interpreted as inferred candidate circuits rather than complete representations of all possible intracellular routes connecting morphogen input to morphogen output.

### Mathematical Models for Regulatory Circuits Between Communicating Cells.

We use reduced ODE models to explore qualitative dynamical behaviors that can be generated by the inferred cell-cell hypermotif topologies. These models are not fitted to kinetic measurements and are not intended to provide quantitative predictions of specific in vivo circuits. Rather, the parameter sets shown in Fig. 4 illustrate distinct dynamic regimes supported by the inferred topologies. For each topology, the parameter values shown were chosen to illustrate representative qualitative regimes, such as pulsatile, oscillatory, antagonistic, or delayed responses, rather than to exhaustively map the full parameter space. Other behaviors may occur under different parameter choices.

Dashed inhibitory arrows in Fig. 4 indicate morphogen antagonism inferred from antagonist expression. In the reduced models, these antagonists are not represented as separate dynamical variables. Instead, their effects are incorporated as changes in effective morphogen availability, turnover, degradation, or direct inhibitory terms in the ligand equations. A more detailed quantitative model would require explicit representation of antagonist production, ligand binding, receptor activation, and diffusion.

We model the dynamical behavior of two morphogen ligands that regulate each other’s expression through regulatory circuits between a pair of communicating cells (Fig. 4).

For the cell circuit depicted in Fig. 4*B* we use the following set of differential equations where x represents the FOS/SMAD1/NR2F2/FOXP2 module, y represents HNF4A, Z is PPARG, u is for IHH, and v represents BMP2/4/5/7:

$$\frac{\mathrm{dx}}{\mathrm{dt}}=\frac{v^{n_{\mathrm{vx}}}}{v^{n_{\mathrm{vx}}}+k_{\mathrm{vx}}^{n_{\mathrm{vx}}}}\frac{x^{n_{\mathrm{xx}}}}{x^{n_{\mathrm{xx}}}+k_{\mathrm{xx}}^{n_{\mathrm{xx}}}}-x,$$

$$\frac{\mathrm{dy}}{\mathrm{dt}}=\frac{k^{n_{\mathrm{xy}}}}{x^{n_{\mathrm{xy}}}+k_{\mathrm{xy}}^{n_{\mathrm{xy}}}}\frac{y^{n_{\mathrm{yy}}}}{y^{n_{\mathrm{yy}}}+k_{\mathrm{yy}}^{n_{\mathrm{yy}}}}-y,$$

$$\frac{\mathrm{dz}}{\mathrm{dt}}=\frac{y^{n_{\mathrm{yz}}}}{y^{n_{\mathrm{yz}}}+k_{\mathrm{yz}}^{n_{\mathrm{yz}}}}-z,$$

$$\frac{\mathrm{du}}{\mathrm{dt}}=\frac{y^{n_{\mathrm{yu}}}}{y^{n_{\mathrm{yu}}}+k_{\mathrm{yu}}^{n_{\mathrm{yu}}}}\frac{z^{n_{\mathrm{zu}}}}{z^{n_{\mathrm{zu}}}+k_{\mathrm{zu}}^{n_{\mathrm{zu}}}}-u,$$

$$\frac{\mathrm{dv}}{\mathrm{dt}}=\frac{u^{n_{\mathrm{uv}}}}{u^{n_{\mathrm{uv}}}+k_{\mathrm{uv}}^{n_{\mathrm{uv}}}}-v.$$

We used the following parameter values for Fig. 4*C*:

$$k_{\mathrm{uv}}=k_{\mathrm{vx}}=k_{\mathrm{xx}}=k_{\mathrm{xy}}=k_{\mathrm{yu}}=k_{\mathrm{yy}}=k_{\mathrm{yz}}=k_{\mathrm{zu}}=0.01,$$

$$n_{\mathrm{uv}}=n_{\mathrm{vx}}=n_{\mathrm{xx}}=n_{\mathrm{xy}}=n_{\mathrm{yu}}=n_{\mathrm{yz}}=n_{\mathrm{zu}}=1,n_{\mathrm{yy}}=3,$$

with the following initial conditions:

$$x_{0}=z_{0}=u_{0}=v_{0}=0.01,\;y_{0}=1.$$

And the following parameter values for Fig. 4*D*:

$$k_{\mathrm{uv}}=k_{\mathrm{xx}}=k_{\mathrm{xy}}=k_{\mathrm{yu}}=k_{\mathrm{yz}}=k_{\mathrm{zu}}=0.01,\;k_{\mathrm{vx}}=4.3,\;k_{\mathrm{yy}}=0.3,$$

$$n_{\mathrm{uv}}=n_{\mathrm{vx}}=n_{\mathrm{xx}}=n_{\mathrm{xy}}=n_{\mathrm{yu}}=n_{\mathrm{yz}}=n_{\mathrm{zu}}=n_{\mathrm{yy}}=1,$$

with the following initial conditions:

$$x_{0}=y_{0}=z_{0}=u_{0}=v_{0}=0.01.$$

For the cell circuit depicted in Fig. 4*E* we use the following set of differential equations where x is IRF1, y is TCF12/4, Z is SOX6, u is for BMP4, and v represents WNT4. To account for effect of expression of BMP and WNT antagonists, we added direct inhibition terms in the equations for the morphogen ligands:

$$\frac{\mathrm{dx}}{\mathrm{dt}}=1-\alpha x,$$

$$\frac{\mathrm{dy}}{\mathrm{dt}}=\frac{v^{n_{\mathrm{vy}}}}{v^{n_{\mathrm{vy}}}+k_{\mathrm{vy}}^{n_{\mathrm{vy}}}}\frac{x^{n_{\mathrm{xy}}}}{x^{n_{\mathrm{xy}}}+k_{\mathrm{xy}}^{n_{\mathrm{xy}}}}\frac{k_{\mathrm{yy}}^{n_{\mathrm{yy}}}}{x^{n_{\mathrm{yy}}}+k_{\mathrm{yy}}^{n_{\mathrm{yy}}}}-y,$$

$$\frac{\mathrm{dz}}{\mathrm{dt}}=\frac{k_{\mathrm{xz}}^{n_{\mathrm{xz}}}}{x^{n_{\mathrm{xz}}}+k_{\mathrm{xz}}^{n_{\mathrm{xz}}}}\frac{k_{\mathrm{yz}}^{n_{yz}}}{y^{n_{\mathrm{yz}}}+k_{\mathrm{yz}}^{n_{\mathrm{yz}}}}\frac{k_{\mathrm{zz}}^{n_{\mathrm{zz}}}}{z^{n_{\mathrm{zz}}}+k_{\mathrm{zz}}^{n_{\mathrm{zz}}}}-z,$$

$$\frac{\mathrm{du}}{\mathrm{dt}}=\frac{u^{n_{\mathrm{uu}}}}{u^{n_{\mathrm{uu}}}+k_{\mathrm{uu}}^{n_{\mathrm{uu}}}}\frac{k_{\mathrm{vu}}^{n_{\mathrm{vu}}}}{v^{n_{\mathrm{vu}}}+k_{\mathrm{vu}}^{n_{\mathrm{vu}}}}\frac{x^{n_{\mathrm{xu}}}}{x^{n_{\mathrm{xu}}}+k_{\mathrm{xu}}^{n_{\mathrm{xu}}}}\frac{k_{\mathrm{zu}}^{n_{\mathrm{zu}}}}{z^{n_{\mathrm{zu}}}+k_{\mathrm{zu}}^{n_{\mathrm{zu}}}}-u,$$

$$\frac{\mathrm{dv}}{\mathrm{dt}}=\frac{k_{\mathrm{uv}}^{n_{\mathrm{uv}}}}{u^{n_{\mathrm{uv}}}+k_{\mathrm{uv}}^{n_{\mathrm{uv}}}}\frac{k_{\mathrm{vv}}^{n_{\mathrm{vv}}}}{v^{n_{\mathrm{vv}}}+k_{\mathrm{vv}}^{n_{\mathrm{vv}}}}-v.$$

We used the following parameter values for Fig. 4*F*:

$$k_{\mathrm{uu}}=k_{\mathrm{vu}}=10.2,\;k_{\mathrm{uv}}=k_{\mathrm{vy}}=k_{\mathrm{xy}}=k_{\mathrm{xz}}=k_{\mathrm{yy}}=k_{\mathrm{yz}}=k_{\mathrm{zz}}=0.01,\;k_{\mathrm{vv}}=4.7,$$

$$k_{\mathrm{xu}}=0.3,\;k_{\mathrm{zu}}=6.3,\;\alpha =5.7,$$

$$\begin{array}{ll} n_{uu} & =n_{uv}=n_{vu}=n_{vy}=n_{xy}=n_{xz}=n_{yy}=n_{yz}=n_{zu}\\ & =n_{zz}=1,\;n_{vv}=n_{xu}=3, \end{array}$$

with the following initial conditions:

$$x_{0}=y_{0}=z_{0}=u_{0}=v_{0}=0.01.$$

And the following parameter values for Fig. 4*G*:

$${k_{\mathrm{uu}}=k}_{\mathrm{uv}}=k_{\mathrm{vy}}=k_{\mathrm{xy}}=k_{\mathrm{xz}}=k_{\mathrm{yy}}=k_{\mathrm{yz}}=k_{\mathrm{zz}}=0.01,$$

$$k_{\mathrm{xu}}=k_{\mathrm{zu}}=k_{\mathrm{vv}}=0.3,\;\alpha =0.01,$$

$$\begin{array}{ll} n_{vv} & =n_{vy}=n_{xy}=n_{xu}=n_{xz}=n_{yy}=n_{yz}=n_{zu}\\ & =n_{zz}=1,\;n_{uu}=n_{vu}=n_{uv}=3, \end{array}$$

with the following initial conditions:

$$x_{0}=y_{0}=z_{0}=v_{0}=0.01,\;u_{0}=0.46.$$

For the cell circuit depicted in Fig. 4*H* we use the following set of differential equations where x is KLF5, y is TCF7L2, w is HNF4A, Z is FOXA1, u is for IHH, and v represents WNT4:

$$\frac{\mathrm{dx}}{\mathrm{dt}}=1-\alpha x,$$

$$\frac{\mathrm{dy}}{\mathrm{dt}}=\frac{v^{n_{\mathrm{vy}}}}{v^{n_{\mathrm{vy}}}+k_{\mathrm{vy}}^{n_{\mathrm{vy}}}}\frac{x^{n_{\mathrm{xy}}}}{x^{n_{\mathrm{xy}}}+k_{\mathrm{xy}}^{n_{\mathrm{xy}}}}\frac{k_{\mathrm{yy}}^{n_{\mathrm{yy}}}}{y^{n_{\mathrm{yy}}}+k_{\mathrm{yy}}^{n_{\mathrm{yy}}}}\frac{w^{n_{\mathrm{wy}}}}{w^{n_{\mathrm{wy}}}+k_{\mathrm{wy}}^{n_{\mathrm{wy}}}}-y,$$

$$\frac{\mathrm{dw}}{\mathrm{dt}}=\frac{x^{n_{\mathrm{xw}}}}{x^{n_{\mathrm{xw}}}+k_{\mathrm{xw}}^{n_{\mathrm{xw}}}}\frac{w^{n_{\mathrm{ww}}}}{w^{n_{\mathrm{ww}}}+k_{\mathrm{ww}}^{n_{\mathrm{ww}}}}\frac{y^{n_{\mathrm{yw}}}}{y^{n_{\mathrm{yw}}}+k_{\mathrm{yw}}^{n_{\mathrm{yw}}}}-w,$$

$$\frac{\mathrm{dz}}{\mathrm{dt}}=\frac{w^{n_{\mathrm{zw}}}}{w^{n_{\mathrm{zw}}}+k_{\mathrm{zw}}^{n_{\mathrm{zw}}}}\frac{k_{\mathrm{zz}}^{n_{\mathrm{zz}}}}{z^{n_{\mathrm{zz}}}+k_{\mathrm{zz}}^{n_{\mathrm{zz}}}}-z,$$

$$\frac{\mathrm{du}}{\mathrm{dt}}=\frac{z^{n_{\mathrm{zu}}}}{z^{n_{\mathrm{zu}}}+k_{\mathrm{zu}}^{n_{\mathrm{zu}}}}\frac{w^{n_{\mathrm{wu}}}}{w^{n_{\mathrm{wu}}}+k_{\mathrm{wu}}^{n_{\mathrm{wu}}}}-u,$$

$$\frac{\mathrm{dv}}{\mathrm{dt}}=\frac{u^{n_{\mathrm{uv}}}}{u^{n_{\mathrm{uv}}}+k_{\mathrm{uv}}^{n_{\mathrm{uv}}}}-v.$$

We used the following parameter values for Fig. 4*I*:

$$k_{\mathrm{ij}}=0.3,\;n_{\mathrm{ij}}=3\;\mathrm{for}\;\mathrm{every}\;i,\;j,$$

with the following initial conditions:

$$x_{0}=u_{0}=v_{0}=0.01,\;y_{0}=1.1,\;z_{0}=0.33,\;w_{0}=0.83.$$

And the following parameter values for Fig. 4*J*:

$n_{\mathrm{ij}}=3$ for every i,j except for $n_{\mathrm{vy}}=1$

$$\begin{array}{ll} k_{uv} & =k_{wu}=k_{ww}=k_{wy}=k_{wz}=k_{xu}=k_{xw}=k_{yw}=k_{zu}=k_{zz}\\ & =0.01,\;k_{vy}=k_{xy}=k_{yy}=0.3, \end{array}$$

with the following initial conditions:

$$x_{0}=y_{0}=w_{0}=z_{0}=v_{0}=u_{0}=0.01.$$

## Supplementary Material

Appendix 01 (PDF)

Dataset S01 (PDF)

Dataset S02 (PDF)

## Data Availability

Previously published data were used for this work (32).
