# Supplementary material for: Regulation of morphogen signaling pathways by recurrent hypermotif circuits
Source: Proc Natl Acad Sci U S A. 2026 Oct 5;123(41):e2538060123. doi: 10.1073/pnas.2538060123 (PMC13663785; doi:10.1073/pnas.2538060123)
Supplement: Supplementary file 1 [file pnas.2538060123.sapp.pdf]

SI Datasets:

Dataset S1: Inferred gene regulatory network edges across intestinal development

Dataset S2: Morphogen pathway gene sets used to quantify morphogen-to-morphogen regulation
