# Supplementary material for: Regulation of morphogen signaling pathways by recurrent hypermotif circuits
Source: Proc Natl Acad Sci U S A. 2026 Oct 5;123(41):e2538060123. doi: 10.1073/pnas.2538060123 (PMC13663785; doi:10.1073/pnas.2538060123)
Supplement: Supplementary file 2 [file pnas.2538060123.sd01.pdf]

Regulating	Target Gen	Sign of Inferred Interaction
PML	PPARG	-1
KLF2	KIF12	-1
IRF1	ETV3	1
IRF7	ALDH1A3	1
DDIT3	BMP5	-1
NFE2L2	RARA	1
KLF5	KIF12	1
KLF4	SOX11	-1
EGR1	HOXA10	1
BCL3	FOXF2	1
FOXF1	TCF4	1
CDX1	RUNX1T1	-1
KLF2	FOXA3	-1
PPARG	ID1	1
ELF3	SOX18	-1
MNX1	KIF5C	1
KLF5	SOX11	-1
EGR1	GLIS3	-1
SOX4	HMGB3	1
ELF3	RUNX1	-1
CEBPB	FOXF1	1
HOXB9	LGR5	1
RREB1	NR2F2	-1
SOX9	SOX9	-1
EGR1	ALDH18A1	-1
JUNB	SOX11	1
PBX1	FGF13	1
MEF2C	ALDH2	-1
JUN	HOXB5	-1
HNF4A	KIF22	1
HES4	HOXA9	-1
SOX4	SFRP1	1
FOS	TBX3	-1
KLF5	BMPR2	-1
MAFF	FOXN3	1
ETV1	ETV1	-1
IRF8	GPC4	1
IRF6	FOXF1	-1
ETS2	RARA	1
GATA6	GATA6	-1
FOXF2	FOXK1	1
ELF3	LEFTY1	1
FOS	RUNX1	-1

KLF5	TCF12	-1
HOXB9	HOXB7	1
PML	HOXB9	-1
SMAD1	SMAD1	1
ESRRA	LRP5	1
FOXO3	TBX3	1
BATF3	GLIS3	1
MNX1	ELF3	1
FOS	GATAD2A	-1
SRF	FGF13	1
EGR1	GLI3	1
GADD45A	SFRP2	-1
HOXA10	HOXA9	1
MYC	ALDH18A1	-1
FOS	BMP2K	-1
EGR1	CRABP2	1
HOXD1	HOXB9	1
HOXB2	HOXB4	1
ELF3	SMAD7	1
ETV6	ETV6	-1
TEAD3	FGFR2	1
CDX1	TCF7L1	-1
CDX1	PTCH1	-1
CDX2	HOXB2	-1
FOXF1	RDH10	1
TEAD1	FGFR2	1
FOSL2	FOXN3	1
FOXA1	SMAD9	-1
ETV6	HOXA10	-1
SOX6	ETV1	1
HOXD1	HOXA5	1
KLF5	FZD2	-1
TGIF2	RORA	1
MYC	SOX4	1
DDIT3	HHIP	-1
HES1	ACVR1	-1
JUN	ID1	1
KLF10	FZD2	1
IRF6	ID4	-1
ATF3	HOXA5	-1
FOXA1	ELF3	1
ETS2	SPATS2	1
JUNB	SOX6	-1
KLF2	FZD2	1

HNF4A	FOXP4	1
TEAD2	RUNX1T1	1
BCL3	HOXA3	1
KLF10	ELF1	-1
MEF2C	HOXB3	1
KLF5	GLI3	-1
PPARG	ALDH1A1	1
HOXD10	TCF21	1
HOXB2	RUNX1T1	1
ELF3	HOXB4	-1
TWIST1	SFRP2	1
HOXA11	TCF21	1
SOX4	TBX3	1
ETS2	TCF4	1
HOXB5	ALDH1A3	1
TRPS1	HOXD1	-1
SIN3A	KIF5C	-1
HOXA10	HOXA11	1
PBX1	FOXA3	-1
CUX1	BMP2K	1
KLF5	HOXB4	-1
NFE2L2	NCOA7	1
BCL3	ETS1	1
IRF7	RXRA	1
CEBPG	HOXA13	1
MEF2C	GATAD2A	1
KLF5	ELF1	1
CEBPB	FGFR2	1
CREB3L1	FOXP1	-1
FOXF1	FOXP2	1
IRF1	ETV5	1
HOXA5	HOXA3	1
KLF2	SFRP1	1
FOXF1	BMP4	1
CDX1	HOXB4	-1
IRF1	RORA	1
CDX2	TCF4	-1
HOXB5	KIF5C	1
NFE2L2	FOXP1	1
FOS	RUNX1T1	1
HOXB9	HOXA7	1
REST	SOX18	1
NR4A1	ETV1	-1
SOX4	SOX13	1

KLF6	LRP1	-1
FOXF2	GPC6	1
HOXA5	HOXB5	1
IRF6	FZD5	1
HNF4A	ALDH18A1	1
MEF2C	ID4	1
EGR1	HHIP	-1
EGR3	SOX5	1
JUN	HOXD10	-1
HOXB5	HOXB5	-1
PBX1	SFRP2	1
HDAC2	HNF4G	-1
EGR1	RUNX1	-1
ESRRA	FOXN2	1
CREM	NR2F2	1
BCL3	PTCH1	1
IRF8	FGFR2	1
RUNX1	RUNX1	1
JUNB	ID3	1
SUZ12	RARS	1
JUN	HOXA9	1
CEBPB	FOXO3	1
IRF1	PTCH1	1
SOX4	HOXB2	1
PRDM1	TBX3	1
FOXP1	NCOR2	-1
FOXA3	FOXA1	1
XBP1	FOXA3	1
SUZ12	HOXA3	1
FOXQ1	FOXF1	1
EGR2	ID3	1
KLF10	RORA	1
CDX1	ELF3	1
IRF8	FOXQ1	1
HES4	APC	-1
FOXF1	SMAD9	1
XBP1	WNT2B	1
FOS	SOX4	1
STAT1	SOX13	1
SOX18	SOX13	1
ESRRA	HOXB2	-1
CEBPA	GLI3	-1
MAFG	RARA	1
KLF5	FZD1	-1

MAFF	SMAD7	1
NR2F2	TBX2	1
NR2F2	KIF5C	-1
HNF4A	PPARG	1
KLF6	BMP4	-1
HNF4A	HOXA9	1
CEBPB	ELK3	-1
MAFF	FOXF1	1
NR2F1	KIF13B	-1
PBX3	WNT2B	1
MYC	IHH	-1
ESRRA	CRABP2	-1
SMARCA4	BMPR2	1
FOS	FOXO3	-1
CREM	SMAD3	1
STAT1	KIF22	1
FOXF2	RDH10	1
XBP1	ETS2	1
XBP1	KIF20B	1
NR2F2	ETS2	-1
HOXD10	SPATS2	1
SOX6	SOX6	1
TEAD2	FGFR2	1
SREBF1	LGR5	1
FOS	TCF21	1
JUN	ETS1	-1
KLF2	TBX2	1
RCOR1	HOXB3	1
SOX18	FOXP1	1
CREM	ETV3	1
KLF6	PTCH1	-1
FOXA3	HOXA3	1
BCL3	ETV6	1
NFE2L2	RDH10	1
HOXB6	HOXB5	1
FOXA3	SOX4	1
HNF4A	ALDH1A1	1
ELF3	PTCH1	-1
MNX1	HNF4A	1
FOXF2	HOXD10	1
SOX5	SOX5	1
SUZ12	KIF22	1
BCL3	RUNX1	1
FOS	HOXB3	1

KLF5	HOXB5	-1
ELK3	ETV6	1
HES1	GPC6	-1
KLF6	AKT3	-1
FOXF1	NCOR2	1
ELK3	ALDH16A1	1
PPARA	PPARA	1
EGR3	FOXO1	1
HES1	RUNX1	-1
HOXA5	HOXA4	1
HOXB9	HOXD1	1
FOSL2	ACVR1B	1
HDAC2	FOXP1	1
NR4A1	KIF5C	-1
MYC	HOXB9	1
MEF2C	SMAD1	1
ATF3	ETV3	1
ELK3	FGFR1	1
FOSL1	FOXO1	1
BCL3	FRZB	1
ELF3	SOX5	-1
EGR1	ID2	1
JUNB	HNF4A	-1
SOX4	FOXA3	1
FOXQ1	HOXA9	1
ATF3	NR2F2	1
HDAC2	KIF1B	1
CDX1	FOXF1	-1
MEF2C	SPATS2L	-1
BHLHE40	HOXB4	1
CEBPB	RXRA	1
HOXA11	HOXA10	1
KLF2	TCF4	1
KLF5	HOXB7	1
NFIL3	BMP2	1
HOXD10	HOXD8	1
FOS	ACVR1B	-1
IRF8	RXRA	1
ELF3	SFRP1	-1
KLF2	TBX3	-1
SUZ12	HMGB3	1
CREB3L1	FGFR1	1
FOS	ID4	1
KLF6	HHIP	-1

SMAD1	SFRP2	1
ATF3	HOXA9	-1
SMARCA4	AKT3	1
HOXA11	HOXA11	1
SOX6	RUNX1	1
ETS2	SOX9	1
ELK3	ETS2	1
ATF3	TBX2	1
FOXA1	FOXA1	1
FOSL2	FZD5	1
ATF3	LRP10	1
EGR2	TCF12	1
PPARG	FZD5	1
FOXP1	TBX2	-1
PML	LGR5	-1
BCL3	ACVR1B	1
IRF1	RUNX1T1	1
IRF1	HOXA13	-1
MEF2C	AKT3	1
TRPS1	ALDH1A1	-1
PBX1	HNF4A	-1
XBP1	ID3	1
KLF5	NR2F2	-1
ELF3	RDH10	-1
IRF8	TCF21	1
KLF2	SOX4	1
CUX1	HOXA10	1
EZH2	KIF20B	1
BCL3	FOXF1	1
NR4A1	TBX3	-1
HNF4A	FLII	1
HOXA11	HOXA9	1
BCL3	BMP2	1
NFIL3	HOXA5	-1
MAF	SOX6	1
IRF1	FOXF2	1
IRF6	IHH	1
FOXF1	GLIS2	1
EGR3	LRP12	1
KLF5	NCOA3	-1
CDX1	HOXB2	-1
ETV5	ETV5	1
JUN	ID4	1
EGR1	FGFR1	1

MEF2A	RUNX1T1	1
ELF3	HOXB3	-1
JUN	HOXB9	1
ELK3	FOXP4	-1
ETS2	SMAD7	1
BCL3	FOXP2	-1
FOS	SFRP2	1
KLF6	FOXF1	-1
HDAC2	ELK4	1
KLF2	SOX11	1
PBX1	FOXA1	-1
CREB3L2	ACVR1	1
KLF2	HOXB4	1
KLF5	SOX9	1
KLF5	FGFR4	1
STAT1	ELF2	1
KLF2	HOXB2	1
PBX1	RUNX1	-1
BCL3	ID4	-1
JUN	HHIP	-1
FOXF1	FOXO3	1
HOXB6	HOXB4	1
SRF	HOXA3	1
ETS2	GATAD2A	1
IRF1	TBX3	-1
NR2F1	FGF9	-1
KLF10	HOXB9	-1
CEBPB	SPATS2L	1
KDM5B	RORA	1
BCL3	GATAD2A	1
JUNB	ID1	1
KLF2	ACVRL1	1
IRF8	RUNX1	1
CEBPA	SOX11	-1
PRDM1	FOXF2	1
RXRA	FZD1	1
PBX3	SMAD4	1
BHLHE40	SMAD7	1
ETS1	FOXO1	1
HOXA5	HOXA5	1
CDX2	FOXF1	-1
KLF5	GPC1	-1
HOXA7	IHH	1
CREB3L1	HOXB4	-1

HOXB5	TBX3	1
HOXA9	HOXB9	1
HOXA10	SFRP2	1
HNF4A	SOX9	1
PBX1	SOX9	-1
PPARG	GLI4	1
SOX18	RARA	1
FOXP1	TCF21	-1
HNF4A	FZD5	1
CEBPB	RARA	1
SOX4	HOXB4	1
CEBPB	RDH10	1
IRF6	WNT2B	-1
CEBPB	TCF4	1
FOXF1	TBX3	1
SOX4	HOXB5	1
MEF2C	FOXO1	1
ETS2	FOXO3	1
FOXA1	HNF4A	1
PRDM1	HOXA3	1
FOXF1	SMAD7	1
FOXA3	HOXB9	1
PML	GATA6	-1
PBX3	NR2F2	1
PRDM1	ALDH1A3	1
NFKB2	FOXP1	1
KLF6	SFRP2	-1
MNX1	SOX9	1
SAP30	HMGB3	1
FOXA1	HOXA10	1
KLF5	GATA6	1
BCL3	SMAD3	1
KLF2	FGFR4	-1
ETS2	LRP10	1
OVOL1	TCF4	-1
SRF	ID4	1
IRF1	BMP4	1
IRF6	ALDH1A1	1
ELK3	SOX4	1
ETV6	RARS	-1
ZEB1	LGR4	-1
ELK3	SMAD1	1
HNF4A	SMAD7	1
HOXB5	HOXB2	1

EGR1	HOXB5	-1
ETS1	HOXA10	-1
CEBPB	NR2F2	1
FOXF1	TCF21	1
HOXB2	FGFR2	1
ATF3	ID3	1
CREB3L1	PPARG	1
PPARG	SOX9	1
KLF5	FZD5	1
HOXA13	KIF13B	1
FOXF1	HOXD10	1
KLF5	PPARG	1
ELK3	AKT3	1
REST	SFRP1	1
EGR3	ACVR1	1
ELF3	ELK3	-1
SOX4	RGMB	1
IRF1	RARA	1
SOX4	ALDH1A3	1
JUNB	ETV1	-1
SOX9	FZD1	-1
NR2F1	ELF3	-1
IRF6	BMP2K	1
STAT1	PTCH1	1
SMAD3	SMAD3	1
HOXA10	FOXA1	1
HES1	ETV1	-1
ATF3	SOX5	1
NR2F2	NR2F2	-1
TCF7L2	TCF7L2	-1
EGR1	SOX4	1
TWIST1	CRABP2	1
HOXB9	FOXQ1	1
MEF2C	SOX18	1
KLF3	ID1	1
KLF5	ID2	1
PBX3	PTCH1	1
IRF8	FGF2	1
CEBPB	BMP2K	-1
MNX1	IHH	1
FOXF2	FOXF2	1
HOXB5	HOXB3	1
STAT1	GPC4	1
KLF5	LRP12	-1

FOS	PPARD	-1
KLF5	IHH	1
HOXB6	HOXB3	1
MAFF	GPC1	1
MAFB	SMAD9	1
KLF6	NR2F2	-1
TEAD1	FOXF2	1
FOXF1	SPATS2L	1
FOXA1	PPARG	1
KLF2	WNT2B	1
HOXD1	LGR5	1
ETS1	RORA	1
JUNB	FGF9	-1
NFE2L2	ALDH7A1	1
HOXD10	GLI3	1
JUNB	HOXA11	1
KLF5	FGF13	-1
NFE2L2	HHIP	1
NFIL3	LRP10	1
SOX4	HOXA4	1
IRF8	SOSTDC1	1
CEBPA	RUNX1T1	-1
HOXA13	HOXA10	1
KLF5	LEFTY1	1
PBX3	NCOR2	1
FOXO3	FOXO3	1
PBX1	ETV1	-1
MEF2C	ELK4	1
ESRRA	SMAD9	-1
TCF4	HOXD1	-1
ELF3	FOXF2	-1
CREB3L1	ID4	-1
PRDM1	SMAD7	1
CEBPG	FGF13	-1
IRF1	TCF12	1
MEF2C	SOX11	1
ETS2	SOX18	1
RUNX1	SOX6	1
FOSL2	ETS2	1
CREB3L1	SMAD1	1
ETS1	GATAD2A	1
MAFF	NR2F2	1
SOX6	SOX6	-1
FOS	ID1	1

FOS	CRABP2	1
FOXF1	FGFR1	1
KLF5	HNF4G	1
EGR1	RDH10	-1
RCOR1	FOXN3	1
PBX1	TBX2	-1
HOXA9	ID1	1
FOXF2	FOXP2	1
KLF3	ID4	-1
FOXA3	CRABP2	-1
ETV6	FOXP1	1
FOXA1	LGR4	1
FOS	HOXB5	-1
STAT1	KIF2A	1
FOXF2	FOXF1	1
IRF1	FZD2	1
DDIT3	ID2	1
PRDM1	FOXF1	1
PPARG	LGR4	1
RCOR1	BMPR1A	1
HOXA5	SOX4	1
IRF1	HOXA3	-1
ETV5	GLIS2	1
MEF2C	CRABP2	-1
HES4	SOX6	-1
ETS2	TBX3	1
CEBPB	ETS2	1
FOS	ID3	1
HES4	HOXB7	-1
CDX2	SOX11	-1
KLF6	TCF4	-1
FOXP2	SOX4	-1
KLF3	FGFR4	1
IRF8	ALDH1A3	1
JUN	RORA	-1
NFE2L2	TCF4	1
HOXB2	SFRP2	1
KLF6	SOX11	-1
SOX18	SOX11	1
JUN	RUNX1	-1
CDX1	SOX5	-1
ETV6	KIFC3	1
FOXA1	FGFR3	1
SRF	GPC3	-1

CREB3L1	TBX3	-1
NFIL3	ALDH1A3	1
FOS	KIF13B	-1
HNF4A	HNF4A	1
HOXB9	HOXA10	1
FOXO3	FOXF1	1
FOXF2	BMP3	-1
KLF5	FOXP4	1
HNF4A	HOXB9	1
IRF6	SOX9	1
DDIT3	HOXB9	1
HOXA4	HOXA6	1
NFIL3	TBX2	1
ETS1	ACVRL1	1
HOXB4	HOXB4	-1
FOSL1	LGR5	1
MAFF	GLIS3	1
EGR1	ACVR1B	-1
KLF5	KIF5C	1
PBX3	LRP12	1
CEBPB	FOXO1	-1
KLF5	FOXF1	-1
STAT1	RXRA	1
CEBPB	HHIP	1
HNF4A	BMP3	1
KLF2	FOXP1	-1
FOXA1	LGR5	1
MEF2C	NCOA7	-1
EZH2	HMGB3	1
GATA6	SOX11	-1
ELF3	FGFR1	-1
ETS2	BMP2	1
IRF8	FRZB	1
JUNB	HOXB2	1
JUN	HOXB4	1
MAFF	HHIP	1
IRF1	GPC4	1
FOXN2	SMAD7	1
PBX3	FOXF1	1
IRF1	HOXD10	1
GATA6	TCF21	-1
KLF6	FRZB	-1
EGR1	SFRP2	1
PPARA	IHH	1

JUNB	ID4	1
CEBPB	PTCH1	1
IRF1	ALDH16A1	1
NFE2L2	FOXO1	1
ELF3	NR2F2	-1
PRDM1	ACVRL1	1
KLF3	PPARG	1
BATF3	ETS2	1
FOXF1	FGFR2	1
KLF4	FOXP2	-1
IRF1	FRZB	1
CEBPG	IHH	1
HOXA10	HOXA10	1
PRDM1	RXRA	1
JUNB	GLI3	1
MEF2A	HOXD10	1
FOXA3	PPARG	1
JUN	NR2F2	1
CEBPA	SOX4	-1
HOXD1	HOXD8	1
NFIL3	ACVR1	1
ETS1	GPC4	1
HNF4A	ELF3	1
KLF10	NR2F2	1
FOXF2	FZD1	1
XBP1	PPARG	1
KLF5	TCF4	-1
FOXF2	HOXA11	1
SOX4	TBX2	1
KLF3	HOXB2	-1
ATF3	ETV5	1
ESRRA	FGFR3	1
KLF10	SOX13	1
CREB3L2	ETV1	1
ELF3	SOX6	1
CEBPB	FOXF2	1
ETS1	SOX11	1
CEBPB	FZD7	-1
KLF4	FGFR1	-1
MYC	NR2F2	1
KLF2	CRABP2	1
FOXF2	SOX6	1
STAT1	KIF20B	1
EGR2	FOXF1	1

SOX4	NR2F2	1
MEF2C	NR2F2	1
TCF21	HOXC6	1
CEBPA	ID4	-1
PRDM1	SOX6	1
EGR1	BMP2K	-1
ETS1	ELK3	1
SOX18	ID3	1
HOXB9	FOXA3	1
THRB	SMAD3	1
ETS2	SMAD1	1
CREB3L1	TCF21	1
KDM5B	FOXO3	1
ETV6	ETS2	1
EGR1	FOXF2	-1
FOXF2	HOXC6	1
CREM	ELF1	1
HNF4A	FGFR3	1
JUN	SMAD3	-1
FOXF2	RXRA	1
SMAD1	TCF12	1
RELB	PTCH1	1
MEF2A	ETS1	1
FOXF1	SOX5	1
KLF2	SOX18	1
PBX3	FZD2	1
CDX1	FGF13	-1
KLF4	ID1	1
EZH2	RORA	1
NFIL3	SOX11	1
NFE2L2	ETS1	1
MAFF	RARA	1
HOXA9	HOXA10	1
KLF5	ETS1	-1
NFIL3	SOX6	-1
GADD45A	ALDH1A1	-1
KLF2	FZD5	-1
KLF10	HOXB5	-1
CDX1	ETV1	-1
IRF1	KIF22	-1
HOXD10	SFRP2	1
ETV6	FOXO1	1
PBX3	TCF21	1
KLF3	FGF13	-1

KLF4	FZD2	-1
CEBPB	ID3	1
ELK3	GABPA	1
NFE2L2	ACVR1	1
IRF6	FZD7	-1
IRF8	RARRES3	1
ELK3	ETS1	1
CREM	RORA	1
FOXF1	RUNX1	1
IRF7	TBX2	1
BCL3	ALDH16A1	1
CDX1	NR2F2	-1
KLF5	FOXP1	1
CREM	ID4	1
SOX18	SOX4	1
XBP1	FOXO3	1
FOXA3	ID2	1
KLF2	FGFR3	-1
HOXA4	HOXA5	1
ELF3	SOX9	1
TWIST1	RUNX1T1	1
KLF5	TBX2	-1
ELF3	ACVRL1	-1
RELB	BMP2	1
SOX5	RUNX1T1	1
JUN	FOXO3	-1
NFIL3	FGF2	1
FOS	RDH10	-1
CUX1	KIF1B	1
FOXF2	SMAD9	1
CREB3L1	GPC3	1
PML	FOXD2	-1
KLF6	ETV1	-1
FOXF2	ACVR1	1
HOXB2	HOXB5	1
CEBPB	RUNX1	1
CDX1	TBX2	-1
ETS2	BMP4	1
ETS2	GLIS2	1
TCF4	FOXA1	-1
HES1	FOXO3	-1
XBP1	RARA	1
NFIL3	HOXB7	-1
MEF2C	SOX5	1

TWIST1	GLI3	1
ETS1	TBX3	1
TCF7	TCF7	1
FOXP1	FOXP1	1
KLF10	LEFTY1	-1
IRF1	SOX9	-1
KLF5	LRP11	1
KDM5B	HOXB5	1
FOXF2	BMP4	1
ELK3	NCOA7	1
MEF2A	ELK3	1
HOXB2	HOXB2	1
TEAD3	FOXP2	1
CEBPB	FZD5	-1
MAFF	GLI3	1
OVOL1	SOX11	-1
IRF1	WNT2B	1
ETV5	TBX2	1
NFIB	TCF4	1
KLF2	GATA6	-1
IRF1	HOXA10	-1
KLF6	CRABP2	-1
CEBPB	TBX2	1
KLF13	ETS1	1
FOXP2	FOXP2	-1
KLF5	ALDH1A3	-1
HNF4A	FGF9	1
IRF1	SMAD3	1
MAFG	HOXB2	-1
IRF1	FGF9	-1
MEF2D	FOXF2	1
ETS2	CTNNBIP1	1
JUN	FOXA3	1
ATF3	FGF7	1
ELF3	SFRP2	-1
IRF8	PPARD	1
RCOR1	FGFR1	1
CEBPB	ETV6	1
ETV6	SOX18	1
HOXA4	HOXA4	1
RREB1	FOXF1	-1
HOXA13	HOXB9	1
IRF1	SMAD9	1
FOXF2	ETV1	1

HOXA5	GATA6	1
FOXA1	FOXA1	-1
KLF2	SOX6	-1
NFIL3	HOXA9	-1
ELF3	GLI3	-1
FOXF2	FOXF2	-1
KLF5	ETV1	-1
KDM5B	SMAD9	1
SREBF1	FGFR1	-1
ELF3	BMP4	-1
SOX11	SOX11	1
CEBPB	ELF2	1
FOS	HOXA10	1
CEBPB	BMP5	1
PRDM1	ETV6	1
JUN	ID3	1
MAFF	KIF1B	1
FOXA1	HOXA13	1
ETS1	TCF4	1
FOS	ID2	1
IRF6	ID1	1
EGR3	LRP6	1
HNF4A	LRP5	1
ETS1	LGR4	-1
KLF5	ETV6	-1
CEBPB	HOXA9	-1
IRF1	FOXP1	-1
JUN	SMAD9	-1
FOS	HOXA7	1
TCF12	ID1	-1
NFIL3	ELF2	1
IRF1	KIF5C	-1
HES4	ALDH2	-1
KLF2	GLI3	1
BHLHE40	FOXO1	1
IRF1	FZD5	-1
FOS	FGFR2	1
PBX3	FOXP2	1
KLF3	FOXN2	1
XBP1	BMP4	1
JUN	SOX4	1
FOSL1	BMP2	1
MYC	SOX9	1
IRF8	TCF4	1

MEF2C	GATA6	-1
IRF8	BMP2	1
KLF2	FGFR1	1
XBP1	FOXN3	1
ATF3	ETV1	-1
IRF1	HOXB9	-1
MYC	RARA	1
FOXN3	FOXN3	-1
HOXA9	FOXA3	1
RUNX1	BMP5	1
KLF5	RORA	-1
KLF10	BMP3	-1
ETS1	FGFR1OP2	1
HNF4A	FOXA1	1
IRF1	ACVR1	1
KLF3	FOXP1	1
CEBPG	FOXN2	1
CEBPG	FOXP1	1
IRF1	HHIP	1
IRF1	BMP5	1
KLF5	TCF7L1	-1
PBX1	TBX3	-1
HNF4A	FOXQ1	1
NFIL3	LGR5	-1
KLF5	SOX4	-1
ELK3	ELK3	1
HOXA9	HOXA9	1
ELF3	TBX3	-1
FOS	HOXB4	1
SOX4	HOXA5	1
ELK3	ETV3	1
HOXD1	HOXD1	1
HNF4A	LEFTY1	1
IRF1	TCF4	1
IRF1	SMAD7	1
FOXO3	ETV1	1
GATA6	RSPO3	-1
ETS1	SOX18	1
MAFF	FOXP2	1
ETS1	BMP2	1
CEBPG	HNF4A	1
ATF3	ID2	1
MEF2C	ETS1	1
BCL3	BMP4	1

KLF6	HOXB5	-1
CEBPA	TBX2	-1
BHLHE40	ACVRL1	1
PRDM1	BMP5	1
FOXO3	FOXF2	1
CEBPG	KIF16B	1
NFE2L2	FOXO3	1
FOXQ1	FOXF2	1
PBX3	CRABP2	1
RXRA	SPATS2L	1
CREM	ETV6	1
KLF2	SOX13	1
IRF1	FGFR3	-1
NFIL3	HOXA10	-1
KLF10	CRABP2	1
ETS2	ETS2	1
EGR1	FOXO1	1
ZFX	FOXO1	1
MNX1	HOXB9	1
KLF5	ELF3	1
HOXA4	HOXB5	1
GATA6	HOXB4	-1
CEBPB	ACVR1B	1
ATF3	ID1	1
ATF3	TBX3	-1
SOX4	SOX4	1
KLF6	FZD2	-1
FOXA1	ALDH1A1	1
HDAC2	KIF22	1
IRF6	ID2	1
ESRRA	FOXA3	1
EGR3	NCOA3	1
SOX4	HOXA10	1
KDM5B	KIF1B	1
MNX1	ID2	1
FOXF1	ETV5	1
XBP1	SPATS2	1
ATF3	NCOA7	1
NFKB2	ROR2	1
TFEB	FGFR4	1
JUNB	LGR5	-1
KLF5	ELK3	-1
ELK3	HOXB4	1
IRF8	SMAD9	1

FOXQ1	FOXQ1	1
FOXA3	ALDH1A1	1
SMARCA4	SMAD9	1
SREBF1	HOXB9	1
FOXA1	IHH	1
CEBPB	ETV1	1
NFIL3	TCF7L2	-1
JUN	RSPO3	1
MAFF	ID4	1
IRF1	KIF12	-1
HOXA7	HOXB9	1
NR2F2	FZD5	-1
ATF3	HOXB5	-1
SOX4	KIF5C	1
ETS1	NCOA1	1
JUN	AKT3	-1
SOX18	ETS1	1
JUN	HOXA10	1
KLF10	BMP4	1
BCL3	FGFR4	1
SMAD1	ELK3	1
ATF3	HMGB3	1
KLF6	RORA	-1
IRF6	HOXA3	1
HOXA13	GATA6	1
IRF8	SMAD7	1
TCF12	TCF12	1
FOS	KIF5C	-1
FOXF1	SOX6	1
KLF5	FOXP2	-1
ETS1	SMAD1	1
HNF4A	TCF7	1
IRF1	ETS1	1
EGR1	ETS2	-1
FOXP1	FGF13	-1
CEBPA	PTCH1	-1
IRF1	GATA6	-1
FOXF2	HHIP	1
IRF8	ELF2	1
KLF4	TBX2	-1
JUN	FGFR1	-1
ATF3	RARS	-1
ATF3	KIF5C	-1
PRDM1	BMPR2	1

NFIL3	ID4	1
SMARCA4	SMAD1	1
FOXA3	IHH	1
HNF4A	LGR5	1
CEBPB	KIF1B	1
MYC	PTCH2	1
NR4A1	FGFR3	-1
KLF10	HOXB7	-1
BCL3	BMPR2	1
FOXF2	PTCH1	1
FOS	ETV1	-1
HES4	ETV1	-1
KLF5	HOXB2	-1
CEBPB	ETV5	1
FOXA1	SOX9	1
JUN	HOXD8	1
IRF1	HOXB4	1
MYC	ETS2	1
SOX11	HOXB3	1
HOXB9	SOX9	1
FOXA3	FGFR3	1
FOS	BMPR2	-1
TCF3	TCF3	1
KLF2	ELK3	1
KDM5B	FZD1	1
ETV6	NCOA1	1
CDX1	SOX11	-1
ELF3	FZD2	-1
PBX3	SFRP2	1
ETV5	KIFC3	1
PKNOX1	RUNX1	1
NFIL3	RARA	1
HOXA11	RUNX1T1	1
ATF3	FOXP1	1
ATF3	FOXA3	1
FOXF2	HOXB3	1
NFIL3	NR2F2	1
PRDM1	GPC4	1
NFE2L2	RUNX1	1
MNX1	HOXD1	1
SOX4	ID3	1
ELK3	SOX18	1
ELK3	BMP2	1
CEBPB	GPC4	1

FOS	HOXB2	1
ELF3	FOXN3	-1
FOS	ETS2	-1
CEBPB	HOXC6	1
MEF2D	HOXD10	1
FOS	KIFC3	-1
FOXF2	SOX5	1
IRF1	FOXN3	1
FOXQ1	HOXA3	1
MAFG	ETV3	1
MEIS3	FOXK1	1
FOXA1	FOXP1	1
FOXF1	ETV1	1
IRF1	ELK3	1
SOX11	GPC4	1
CEBPG	BMP5	-1
ETV6	RARA	1
FOXO3	HHIP	1
TAF1	HOXC6	1
FOSL2	RORA	1
PBX3	SMAD1	1
TEAD2	NR2F2	1
EGR1	ID3	1
HOXA9	SOX11	-1
MEF2C	HOXB4	1
MSX1	HOXA13	-1
IRF1	FOXD2	-1
SRF	TCF21	-1
NR4A1	KIF21A	-1
FOXF2	WNT2B	1
KLF4	RUNX1T1	-1
ETV5	ELK3	1
HOXB5	HOXB4	1
FOXN2	SMAD1	1
IRF8	ETV1	1
ELF3	TCF21	-1
KLF3	HOXB7	1
ETS2	HOXA3	1
FOXF2	SOSTDC1	1
MEF2A	HOXB3	1
FOXF2	FOXQ1	1
RARA	FOXO1	1
KLF5	FOXF2	-1
CEBPB	NCOA1	1

FOSL2	ACVRL1	1
ELF3	KIF5C	1
IRF1	BMPR2	1
FOXA1	HOXB9	1
SOX4	FOXQ1	1
FOXA1	KIF5C	1
HNF4A	HNF4A	-1
HOXB5	TCF7L2	1
IRF6	SOX4	-1
MEF2C	TBX2	1
HOXA11	SFRP2	1
HDAC2	SMAD5	1
FOS	RXRA	-1
KLF2	SMAD9	1
CEBPG	SOX11	-1
JUN	SOX18	1
PBX3	LRP1	1
SMARCA4	TBX2	1
CEBPB	FZD1	1
EGR1	GATAD2A	-1
ELF3	FOXA3	1
FOXF2	BMP5	1
KLF6	NCOR2	-1
IRF1	ID3	1
PRDM1	LRP10	1
RARA	RARA	1
EGR1	LEFTY1	-1
IRF7	ELF1	1
PPARA	FOXO3	1
ELF3	NCOA3	-1
FOS	HOXA11	1
ATF3	HOXB9	1
IRF1	HOXB2	1
ZEB1	FOXP4	-1
RELB	RORA	1
NFIL3	FOXN3	1
IRF7	RUNX1	1
IRF1	BMPR1A	1
HOXB6	HOXB9	1
KLF2	RORA	1
PBX3	FZD7	1
HOXD1	BMP2	1
NR4A1	ELF3	-1
ZFX	LRP6	1

SREBF1	HNF4A	1
KLF5	AKT3	-1
FOXA1	SOX6	1
PBX3	FGF13	1
ZFX	BMPR1A	1
HOXA7	HOXA7	1
NFIL3	FGFR2	1
EGR3	SOX11	1
TGIF2	ETV5	1
PML	KIF5C	-1
DDIT3	NCOR1	1
CEBPB	FGFR1	1
FOXF1	FZD1	1
PBX3	HHIP	1
CEBPB	SMAD7	1
ETV6	LRP10	1
ETS2	PPARA	1
CEBPB	BMPR2	1
BCL3	RORA	1
CDX2	RUNX1T1	-1
SOX18	ETV6	1
FOSL2	PPARD	1
CEBPB	ALDH1A1	-1
JUN	KIF2A	-1
KLF2	NR2F2	1
FOXQ1	SOX6	1
RELB	SFRP1	1
FOS	ETS1	-1
FOSL2	LEFTY1	1
MAFF	ETV6	1
NFE2L2	RORA	1
NFIL3	HOXB9	-1
ELF3	ETV1	-1
JUN	NCOA1	-1
HNF4A	FGFR4	1
FOXF1	PTCH1	1
HOXA10	SOX4	1
KLF5	FGFR1	-1
SOX18	ACVRL1	1
CEBPB	ID2	-1
KLF2	RUNX1T1	1
PBX1	IHH	-1
JUN	TCF21	1
FOXP2	BMP5	-1

HNF4A	BMP2K	1
MAF	FOXP1	-1
IRF1	ROR2	1
IRF1	IHH	-1
NR2F2	HNF4A	-1
JUN	PPARD	-1
SOX4	LRP12	1
FOXO3	FGFR2	1
XBP1	RXRA	1
CEBPB	HOXA5	-1
HOXA9	HOXA5	1
HOXA5	TBX3	1
FOXA1	HNF4G	1
KLF4	SMAD9	-1
IRF6	GATA6	1
SOX4	GSK3B	1
CDX1	ETV5	-1
CEBPB	GLIS3	1
IRF1	NR2F2	1
HNF4A	ACVR1B	1
ATF3	ETV6	1
ETV5	NCOA2	1
JUN	HOXB2	1
IRF8	FOXF1	1
ETS1	HHIP	1
NFIL3	HNF4A	-1
NFIL3	HOXB3	1
EGR1	HOXB2	1
SOX11	ETS1	1
SOX11	SOX12	1
HOXB5	HOXB6	1
FOXF2	RUNX1	1
SOX4	AKT3	1
FOXP1	HOXB5	-1
PPARG	FOXP1	1
IRF8	FOXF2	1
KLF2	BMP4	1
TCF12	HNF4A	-1
IRF8	HHIP	1
FOXF1	FOXF1	1
KLF3	GATA6	1
KLF5	FZD7	-1
FOS	SOX6	-1
KLF5	WNT2B	-1

KLF3	SMAD7	1
FOXQ1	BMP5	1
EGR1	TCF21	1
STAT1	BMP4	1
PBX1	HOXA5	-1
ETS2	RUNX1	1
ETS1	GPC3	-1
KLF4	ETV1	-1
CREB3L1	SFRP2	1
JUN	KIF5C	-1
ELF3	FOXP1	1
RCOR1	IHH	-1
KLF3	HOXD1	1
RORA	RORA	1
ETV5	SOX11	1
MNX1	HOXA13	1
FOXP2	GATA6	-1
TEAD1	SMAD9	1
ELF3	FOXD2	1
ETS1	LRP12	1
SMAD1	SOX18	1
CEBPB	LRP1	1
RCOR1	SOX4	-1
ELF3	FOXA1	1
KLF4	ELF1	1
MEF2C	SOX6	-1
HOXB5	ETV1	1
EGR1	NR2F2	1
CEBPB	FGFR4	1
SOX11	SOX5	1
SOX6	BMP4	1
XBP1	RDH10	1
GATA6	ID4	-1
FOXP1	FOXP1	-1
HOXB9	HOXA9	1
KLF4	LRP10	1
CEBPB	FOXQ1	1
ATF3	FGFR3	-1
KLF2	ETV5	1
PRDM1	HHIP	1
HOXA13	HOXA13	1
KLF6	TCF21	-1
KLF13	TCF21	-1
JUN	SFRP2	1

JUN	RUNX1T1	1
MNX1	GATA6	1
KLF5	NCOR2	-1
FOXQ1	SOX9	1
ELF3	RORA	-1
HNF4A	PPARA	1
FOXA3	SOX9	1
SAP30	TBX3	1
IRF1	SOX11	1
FOS	NR2F2	1
PPARG	IHH	1
SOX9	NR2F2	-1
CEBPB	BMP2	1
PBX3	HOXA10	1
EGR1	BMPR2	-1
PBX3	FGFR1	1
MAFF	RUNX1	1
ELK3	TCF4	1
ETS1	ETV3	1
EGR3	TBX2	1
BATF3	RDH10	1
ETV6	ETV6	1
FOXP1	GPC6	-1
NFIL3	SPATS2	1
HOXA4	SOX4	1
ETS2	ELF2	1
REST	FRZB	1
KLF2	PPARG	-1
HNF4A	KIF13B	1
HNF4A	LGR4	1
KLF5	HOXB9	1
FOXF1	FOXF2	1
IRF6	HNF4G	1
EGR3	SOX18	1
SOX4	HOXB3	1
MYC	RUNX1	1
HNF4A	ALDH1B1	1
HOXB9	FOXA1	1
SOX4	LGR5	1
HES4	HOXB9	-1
SMARCA4	PAXIP1	1
JUNB	HHIP	-1
FOXF2	TCF21	1
KLF3	HOXB9	1

HES4	BMP4	-1
SOX5	TBX3	1
ELK3	FOXO1	1
ELK3	ACVRL1	1
NFE2L2	ETS2	1
PRDM1	ETS1	1
FOXF2	TBX2	1
FOXA3	FOXP4	1
IRF1	ID2	-1
DDIT3	FOXP1	1
CEBPB	TCF21	1
EGR2	TCF21	1
IRF1	SFRP2	1
IRF1	SPATS2	1
BCL3	SPATS2	1
FOXA1	ACVRL1	-1
BCL3	TBX2	1
MAFF	FGF7	1
KLF5	FOXA1	1
MAFF	BMPR1A	1
KLF5	ID1	1
HOXA9	FOXQ1	1
FOXO3	TCF4	1
IRF1	TCF21	1
HES4	HOXD10	-1
FOS	RORA	-1
JUNB	HOXB5	-1
JUN	ETV1	-1
MYC	RGMB	1
IRF8	GLIS3	1
KLF10	FOXP2	1
MNX1	HOXA9	1
CREM	HOXA10	-1
IRF1	FOXF1	1
TWIST1	BMP4	1
HNF4A	RDH13	1
MYC	FGF7	1
FOSL2	PPARG	1
HOXB5	HOXB5	1
ELK3	ELK3	-1
HOXA9	HOXA9	-1
FOXQ1	BMP4	1
HOXA10	SOX12	1
TEAD2	SOX12	1

HOXA5	ETV1	1
EGR1	SOX6	-1
FOXF1	BMP5	1
EZH2	LRP12	1
ETS2	RXRA	1
RXRA	FOXF1	1
ELK3	HOXD8	1
IRF1	LRP10	1
ATF3	RUNX1	1
ELF3	FZD5	1
NR2F2	HOXA3	-1
JUNB	RUNX1T1	1
FOXA3	FOXQ1	1
IRF1	SOX4	-1
ELF3	KIF9	1
SOX6	TBX3	1
IRF1	SOX13	1
KLF5	KIF13B	1
HOXA4	HOXB3	1
CREM	TCF4	1
KLF5	BMP4	-1
KLF4	FOXF2	-1
HIF1A	ETS1	1
KLF6	WNT2B	-1
HOXD1	TBX3	1
ESRRA	KIF12	1
MEF2D	RARA	1
BCL3	BMP5	1
NFIL3	TCF21	1
JUNB	SFRP2	1
ELF3	FOXP4	1
EGR1	TBX3	-1
ETV6	TCF4	1
ATF3	SOX6	-1
HNF4A	ELF1	1
RXRA	RXRA	1
CEBPG	KIF13B	1
JUNB	NR2F2	1
HOXB5	FGF13	1
NR4A1	SOX6	-1
HOXA5	HOXD1	1
KLF5	KIF1C	1
ETS2	ELK3	1
IRF8	BMP5	1

CREB3L1	ACVR1B	1
EZH2	HNF4G	-1
CEBPB	ROR2	1
KLF2	HOXA3	-1
HDAC2	KIF3A	1
KLF3	ELF1	1
CEBPA	SPATS2L	-1
PPARG	LEFTY1	1
MEF2C	TCF4	1
ATF3	BMP2	1
IRF1	RUNX1	1
CREB3L1	GPC6	1
HOXA10	FOXP2	1
IRF7	FRZB	1
TEAD2	HOXB4	1
PRDM1	SMAD9	1
HOXB9	HNF4G	1
HNF4A	TCF7L2	1
STAT1	GATAD2A	1
TFEB	HNF4G	1
IRF1	ETV6	1
HOXD1	SOX9	1
REST	DKK3	1
SOX5	SOX11	1
CDX1	ID4	-1
BCL3	ACVR1	1
IRF1	APCDD1	1
STAT1	ETS1	1
HOXB6	HOXB6	1
TCF12	TCF12	-1
FOXA1	HOXB7	1
MAFF	ID3	1
HDAC2	AKT3	1
RORA	NCOA3	1
MAFF	SOX11	1
ATF3	ETS2	1
KLF4	FOXN2	1
PBX1	BMP2	-1
KLF5	SMAD9	-1
KLF5	SMAD7	1
FOXA1	ID2	1
FOSL2	BMP2	1
ATF3	RDH13	1
HNF4A	GPC3	1

OVOL1	ID4	-1
KLF4	PTCH1	-1
FOXA3	KIF5C	1
ETS2	SOX6	1
CEBPB	ALDH1A3	1
FOXF1	FOXQ1	1
PBX3	HOXD10	1
ETS1	BMPR2	1
FOXP1	TBX3	-1
BATF3	FOXO3	1
ELK3	HOXA10	-1
NR2F1	SMAD7	-1
MAF	ID3	-1
HNF4A	ID2	1
SMARCA4	KIF5C	1
IRF1	SOX18	1
SOX4	KIF1B	1
TFEB	FGFR3	1
SOX4	ID4	1
FOXF1	HHIP	1
IRF1	SOX5	1
PRDM1	RARRES3	1
DDIT3	FOXF2	-1
FOXF2	FGFR2	1
EGR1	ETS1	-1
JUNB	BMP1	1
FOXO3	RXRA	1
JUNB	KIF5C	-1
PBX1	ID1	-1
FOS	SOX11	1
NFKB2	ACVRL1	1
CEBPG	PPARG	1
CEBPB	KIF5C	-1
GADD45A	NR2F2	-1
FOS	GLI3	1
JUN	FOXF1	-1
IRF1	LEFTY1	-1
MNX1	LEFTY1	1
FOXN3	FOXP2	1
RREB1	HOXB2	-1
NFE2L2	GLIS3	1
SMARCA4	TCF3	1
HOXA11	FZD1	1
MEF2C	KIF5C	-1

NFIL3	HOXB5	-1
HOXB6	HOXA5	1
KLF10	PTCH1	1
IRF1	RARS	-1
KLF5	KIF16B	1
SUZ12	KIF20B	1
FOXA3	HNF4A	1
CEBPB	FOXP1	-1
FOXQ1	LGR5	1
FOXN3	SOX4	-1
FOXF2	FOXO3	1
FOXF1	ACVR1	1
FOS	HHIP	-1
HOXB5	HOXA5	1
FOXQ1	BMP3	1
JUN	HOXB3	1
MEF2C	FOXP2	1
ETV5	NR2F2	1
ETV6	HOXA5	-1
MEF2A	SMAD1	1
KLF5	HNF4A	1
IRF8	PTCH1	1
ESRRA	ETV6	-1
EGR1	RUNX1T1	1
MEF2C	SOX4	1
KLF5	RUNX1T1	-1
CEBPB	FOXN3	1
HOXA4	TBX3	1
FOXF2	SMAD7	1
HOXB5	TBX2	1
MAFF	FOXO3	1
CEBPB	WNT2B	1
HES4	PTCH1	-1
FOXO3	ETS2	1
SMARCA4	HMGB3	1
ATF3	ID4	1
PBX1	TCF21	1
ETS1	ETS1	1
KLF6	FOXF2	-1
MSX1	ID4	-1
ELF3	FOXP2	-1
FOXF1	GPC6	1
SMAD1	ELF1	1
KLF2	FOXO1	1

CDX1	TCF4	-1
HOXB6	LGR5	1
KLF5	FOXN2	1
KLF5	TCF7L2	1
KLF13	TBX2	1
NR2F2	ID4	1
KLF5	HOXA10	1
IRF1	SOX12	1
ELF3	SOX4	-1
RARA	RARA	-1
MYC	FOXQ1	1
IRF1	KIF13B	-1
KLF5	CRABP2	-1
KLF5	HOXA3	1
KLF13	RARA	1
JUNB	ETS1	-1
NFIL3	GLI3	1
SOX18	SMAD7	1
FOXA3	FZD5	1
IRF8	BMP4	1
HOXA9	TCF7L2	1
CEBPB	ID4	1
ZFX	FZD7	1
MAF	SOX9	-1
CREM	TBX2	1
MEF2A	FGF9	-1
PBX1	FOXQ1	-1
ETS2	ETV6	1
NFKB2	ETS1	1
FOXA1	ID1	1
BCL3	ETS2	1
GATA6	FOXF1	-1
NR4A1	FZD5	-1
FOXP1	FZD1	-1
ESRRA	SFRP1	-1
ELF3	HOXA10	1
HNF4A	GATA6	1
TWIST1	LRP12	1
FOXF1	FOXO1	-1
ATF3	SPATS2L	1
IRF6	KIF5C	1
SAP30	HOXB2	1
KLF5	FGFR3	1
KLF5	FOXA3	1

ETV6	HOXB4	1
SOX4	LRP6	1
FOXF1	RXRA	1
RELB	ETS1	1
NR2F2	SOX18	1
JUN	TBX3	-1
FOSL1	ETS2	1
RARA	GPC1	1
NFIL3	GPC6	1
CEBPG	TCF7L2	1
PRDM1	RUNX1	1
ETS1	ETV1	1
NFE2L2	BMPR1A	1
MYC	SFRP1	1
ELF3	RUNX1T1	-1
PRDM1	TBX2	1
ELK3	CTNNBIP1	1
MEF2A	FOXF2	1
EGR1	ETV1	-1
PBX1	FOXO1	-1
HOXB6	SOX9	1
FOXP1	BMP4	-1
ELF3	FZD1	-1
BCL3	ETV3	1
CREB3L1	HOXA11	1
ELF3	FGF13	-1
HDAC2	KIF5C	1
BCL3	FGF13	-1
PPARG	PPARG	1
MNX1	FOXA1	1
EGR1	ID4	1
HOXD10	HOXD10	1
SRF	RARA	1
SMAD1	ETS1	1
IRF1	ACVRL1	1
KDM5B	FZD7	1
HOXB9	HOXB9	1
TAF1	KIF1B	1
ETS1	RARA	1
EZH2	KIF2A	1
ELF3	HOXA3	1
NR2F2	NR2F2	1
IRF7	RORA	1
FOS	FOXF2	-1

REST	NCOA3	1
HNF4A	IHH	1
KLF6	FZD7	-1
HOXB5	FOXP1	-1
IRF1	ALDH1A3	1
SMAD1	CTNNBIP1	1
CEBPB	SOX4	-1
ETS2	FOXF2	1
PPARG	KIF5C	1
CEBPB	BMP4	1
ELF3	TCF4	-1
EGR1	ELF1	-1
HNF4A	HOXA3	1
SOX4	FZD1	1
CEBPA	SOX5	-1
FOXF2	TCF4	1
FOXN2	FOXN2	1
JUN	KIF1B	-1
HOXB9	ELF3	1
ATF3	TCF7L2	-1
IRF1	TBX2	1
KLF6	FOXP2	-1
IRF6	RUNX1T1	-1
FOXF1	FOXF1	-1
HNF4A	NCOA7	1
NR4A1	HHIP	-1
CEBPB	FRZB	1
ELK3	NCOA1	1
JUN	RDH10	-1
TFEB	SOX6	1
HOXB2	SOX4	1
MYC	LGR5	1
IRF1	GLI3	1
KLF5	ACVR1	-1
CEBPB	RORA	1
IRF1	ID4	1
ELF3	ETS1	-1
SOX4	SOX5	1
PPARG	ELF1	1
KLF13	SOX11	1
