# Supplementary material for: Regulation of morphogen signaling pathways by recurrent hypermotif circuits
Source: Proc Natl Acad Sci U S A. 2026 Oct 5;123(41):e2538060123. doi: 10.1073/pnas.2538060123 (PMC13663785; doi:10.1073/pnas.2538060123)
Supplement: Supplementary file 3 [file pnas.2538060123.sd02.pdf]

**BMP**	SMAD1	SMAD3	SMAD4	SMAD5	SMAD6	SMAD7	SMAD9	
**WNT**	TCF12	TCF15	TCF19	TCF20	TCF21	TCF3	TCF4	TCF7
**FGF**	EHF	ELF1	ELF2	ELF3	ELF4	ELK3	ELK4	ERG
**HH**	GLI2	GLI3	GLIS2	GLIS3				
**Retinoic Acid**	NCOA1	NCOA2	NCOA3	NCOA4	NCOA6	NCOA7	NCOR1	NCOR2

TCF7L1	TCF7L2	TCFL5	LEF1					
ETS1	ETS2	ETV1	ETV3	ETV4	ETV5	ETV6	FLI1	GABPA
RARA	RARB	RARG	RARRES1	RORA	RXRA	STAT1	STAT6	

**BMP**	BMP1	BMP10	BMP15	BMP2	BMP2K	BMP3	BMP4	BMP5	BMP6
**WNT**	DKK1	DKK2	DKK3	DKK4	RSPO1	RSPO2	RSPO3	RSPO4	SFRP1
**FGF**	FGF1	FGF10	FGF11	FGF12	FGF13	FGF14	FGF16	FGF17	FGF18
**HH**	DHH	IHH	SHH						
**Retinoic A**	ALDH1A1	ALDH1A2	ALDH1A3	ALDH1A7	RARRES2	RDH10			

BMP7	BMP8A	BMP8B	CER1	LEFTY1	LEFTY2	RGMA	RGMB	SOST	SOSTDC1
SFRP2	WIF1	WNT1	WNT10A	WNT10B	WNT11	WNT16	WNT2	WNT2B	WNT3
FGF19	FGF2	FGF20	FGF21	FGF22	FGF23	FGF3	FGF4	FGF5	FGF6

WNT3A	WNT4	WNT5A	WNT5B	WNT6	WNT7A	WNT7B	WNT8A	WNT8B	WNT9A
FGF7	FGF8	FGF9							

WNT9B

**BMP**	ACVR2A	ACVR2B	BMPR1A	BMPR1B	BMPR2	ENG	RGMA	RGMB	
**WNT**	FZD1	FZD10	FZD2	FZD3	FZD4	FZD5	FZD6	FZD7	FZD8
**FGF**	FGFR1	FGFR1OP	FGFR1OP2	FGFR2	FGFR3	FGFR4	FGFRL1	GPC1	GPC2
**HH**	PTCH1	PTCH2	PTCHD1	PTCHD2	SMO				
**Retinoic A**	NCOA1	NCOA2	NCOA3	NCOA4	NCOA5	NCOA6	NCOA7	NCOR1	NCOR2

FZD9	LGR4	LGR5	LGR6	LRP5	LRP6	ROR1	ROR2	RSPO1	RSPO3
GPC3	GPC4	GPC5	GPC6						
RARA	RARB	RARG	RARRES1	RARRES2	RORA	RORB	RORC	RXRA	RXRB

RSPO4 RYK

RXRG

**BMP**	NOG	CHRD	CHRDL1	CHRDL2	FSTL1	FSTL5	FSTL4	FSTL3	TWSG1
**WNT**	SFRP2	SFRP4	SFRP1	SFRP5	FRZB	WIF1	DKK2	DKK4	DKK3
**FGF**	SPRY1	SPRY2	SPRY3	SPRY4					
**HH**	HHIPL2	HHIP.AS1	HHIP	HHIPL1					
**Retinoic Acid**									

NBL1	GREM2	GREM1	DAND5	CER1	SOST	SOSTDC1
DKK1	DKKL1					
